# Enhancing Exercise Performance with Natural Products: A Comprehensive Review

**DOI:** 10.3390/nu18152450

**Published:** 2026-07-27

**Authors:** Amama Rani, Sojin Kang, Sojin Kim, Jung-Hyun Kim, Jimin Kim, Muhammad Alaa Eldeen, Eunbin Ko, Chanju Im, Moon Nyeo Park, Yunju Jeong, Byung-Kwan Seo, Hyun Chul Jung, Bonglee Kim

**Affiliations:** 1 Department of Pathology, College of Korean Medicine, Kyung Hee University, Seoul 02447, Republic of Korea; amama.rani@khu.ac.kr (A.R.); bmb1994@khu.ac.kr (S.K.); dr.muhammadalaa@gmail.com (M.A.E.); mnpark@khu.ac.kr (M.N.P.); 2 Department of Microbiology, Quaid-i-Azam University, Islamabad 45320, Pakistan; 3 Sports Medicine and Science, Graduate School of Physical Education, Kyung Hee University, Yongin-si 17104, Republic of Korea; sojin2150@khu.ac.kr (S.K.); jimin8902@khu.ac.kr (J.K.); 4 Department of Acupuncture and Moxibustion Medicine, Kyung Hee University Hospital at Gangdong, Seoul 05278, Republic of Korea; dan_mi725@naver.com (J.-H.K.); seohbk@hanmail.net (B.-K.S.); 5 Cell Biology, Histology and Genetics Division, Zoology Department, Faculty of Science, Zagazig University, Zagazig 44519, Egypt; 6 Department of Clinical Korean Medicine, Graduate School, Kyung Hee University, Seoul 02447, Republic of Korea; keb26105@naver.com (E.K.); ckswn103@naver.com (C.I.); 7 Department of Food and Nutrition, Kyung Hee University, Seoul 02447, Republic of Korea; yjeong24@khu.ac.kr; 8 Department of Sports Coaching, College of Physical Education, Kyung Hee University, Yongin-si 17104, Republic of Korea; 9 Dr. B Lab Co., Ltd. Room 513, Samuiwon Startup Center, Kyung Hee University, Seoul 02447, Republic of Korea

**Keywords:** natural products, exercise performance, endurance, herbal supplements, exercise-induced fatigue

## Abstract

Background/Objectives: Physical exercise has been a cornerstone of health and vitality across all age groups; however, while its importance remains undeniable, the complications associated with exercise such as fatigue, muscle damage, inflammation, and oxidative stress are not always effectively managed through conventional strategies. This review aims to highlight the current evidence on the potential role of natural products in alleviating these exercise-induced responses and supporting exercise performance and recovery. Methods: The available preclinical and clinical literature was comprehensively reviewed to evaluate the effects of natural products on exercise performance and recovery. Evidence was categorized according to five major mechanisms: attenuation of muscle fatigue, enhancement of muscular endurance, improvement of muscular strength and neuromuscular function, modulation of inflammation and other physiological benefits. Results: Approximately 164 natural products have been investigated for their potential to improve exercise performance and recovery by targeting fatigue-related markers, improving energy metabolism, and regulating oxidative stress. Conclusions: These findings underscore the potential of natural products as promising agents as complementary strategies for addressing exercise-induced fatigue and improving recovery. Although current findings indicate biological plausibility and therapeutic potential of natural products, further clinical evidence is required before definitive performance related recommendations can be established. Future well-designed clinical trials are essential to establish efficacy, safety and translational applicability.

## 1. Introduction

Physical exercise has played a central role in human health since early civilization. Evidence from 3000 BCE indicates that nomadic societies relied on movement for survival, and over time, activity evolved into culturally embedded practices such as games, rituals, and dance [1]. Classical physicians, including Hippocrates and Galen, emphasized exercise as a vital component of health maintenance and coined concepts such as “exercise is medicine” [2]. In modern society, structured exercise remains essential, with the COVID-19 pandemic further highlighting its role in immune function and overall well-being [3].

Caspersen distinguished “physical activity” as any energy-expenditure movement and “exercise” as structured, planned, and repetitive activity aimed at improving fitness [4]. This distinction is particularly relevant for intervention-based research, as “exercise” denotes controlled, fitness-oriented training widely used in experimental and clinical studies (e.g., over 19,882 citations on Google Scholar). Despite global recommendations, physical inactivity remains a major public health challenge. The WHO Global Action Plan on Physical Activity (GAPPA 2018–2030) targets a 15% reduction by 2030; however, worldwide inactivity increased from 27.5% in 2016 to 31.3% in 2022, particularly among women and older adults [5,6].

Exercise confers benefits across the lifespan. In older adults, it mitigates frailty [7], sarcopenia [8], cardiovascular dysfunction [9], and enhances cognitive function [10]; accordingly, WHO guidelines emphasize aerobic, resistance, and balance training to preserve muscle mass, mobility, and independence [11,12]. In children and young adults, ≥60 min of daily moderate-to-vigorous physical activity supports cardiometabolic health, bone development, cognition, and overall well-being [13,14]. Athletes and tactical populations engage in high levels of physical activity and require substantial physical and psychological capacity for optimal performance and public safety [15,16]. Training is periodized into pre-season conditioning, in-season maintenance, and off-season recovery phases to optimize adaptation and minimize injury and fatigue [17].

Exercise interventions spanning aerobic, resistance, and flexibility training improve health but also pose risks when improperly prescribed, such as strains, sprains, tendinopathies, fatigue, and exercise-related physiological stress [18,19]. Pharmacological agents such as anabolic steroids and recombinant human erythropoietin (rHuEPO) may enhance performance but carry significant health risks [20], whereas non-pharmacological therapies like compression garments, cryotherapy, and electrical muscle stimulation (EMS) are used to manage fatigue and aid recovery, although their efficacy varies [21]. Classical massage [22], stretching, and yoga support recovery [23], especially in older adults and athletes, but long-term effectiveness is limited for elite athletes [24]. Consequently, attention has shifted toward safer biological approaches. Overall, conventional approaches are limited by safety concerns, inconsistent outcomes, and a lack of long-term evidence. 

Natural products, particularly plant-derived compounds, have long been used in medicine but remain incompletely explored in exercise science [25]. Derived from bark, leaves, roots, seeds, and fruits, these supplements are widely consumed to enhance physical performance, although mechanistic validation is still emerging [26]. Bioactive metabolites such as alkaloids and phenolic compounds exert anti-inflammatory and vasodilatory effects that support endurance, strength, and recovery [27]. The growing use of herbal adaptogens in health and performance enhancement parallels the rapid expansion of the global herbal market, which is projected to reach USD 347.5 billion by 2029 [28].

High-intensity exercise induces lactic acid accumulation, oxidative stress, and energy depletion, contributing to muscle fatigue [29]. Recovery involves pathways such as AMPK, PI3K/Akt, MAPK, NF-κB, Nrf2/ARE, and PINK1/Parkin, which regulate metabolism, mitochondrial quality control, inflammation, and neuromuscular protection [30]. Natural products modulate key regulators, including PGC-1α, PPARα, mTOR, and Nrf2 to enhance glycogen storage, reduce oxidative stress, and promote muscle adaptation [31,32].

Despite growing interest in natural products for exercise performance and recovery, the literature remains fragmented, with many studies emphasizing individual compounds or outcomes without integrating exercise-induced problems, signaling pathways, and translational relevance. Moreover, comparisons across botanical classes and the molecular networks regulating energy metabolism, oxidative stress, inflammation, and mitochondrial function during exercise are not comprehensively integrated into a unified framework. This review mechanistically examines natural products in the context of exercise, highlighting their roles in reducing fatigue, promoting recovery, and enhancing performance by linking them to key metabolic, inflammatory, and mitochondrial pathways involved in exercise adaptation.

## 2. Methodology

Research articles investigating the effects of natural products on exercise performance and recovery published during the past decade were identified through searches of PubMed and Google Scholar. The search strategy included combinations of keywords related to natural products and exercise outcomes, including “natural products”, “herbal extracts”, “plant-derived compounds”, “exercise performance”, “endurance”, “muscle strength”, “fatigue”, “recovery”, “oxidative stress”, and “inflammation”. Boolean operators (AND/OR) were applied to combine relevant terms and identify eligible studies.

Studies were included if they investigated the effects of natural products on exercise-related outcomes, including fatigue resistance, endurance capacity, muscle strength, muscle recovery, oxidative stress regulation, and inflammatory responses. Eligible interventions comprised herbal extracts, isolated bioactive compounds, decoctions, polysaccharides, vitamins, and other naturally derived products. Both preclinical studies (in vitro and animal models) and clinical studies were included to provide a comprehensive overview of mechanistic insights and translational evidence. Studies were excluded if they were review articles, duplicate publications, conference abstracts lacking sufficient experimental information, or studies unrelated to effects of natural products on exercise performance, fatigue, recovery, or associated physiological mechanisms. Eligible studies were screened manually through title and abstract evaluation, followed by full-text assessment based on the predefined inclusion and exclusion criteria. Data extracted from selected studies included natural product type, source, experimental model, dosage, intervention duration, measured outcomes, and molecular mechanisms. Findings were categorized by functional effects including fatigue reduction, endurance, muscle strength, neuromuscular function, and other physiological responses. As this study was conducted as a comprehensive narrative review, a formal risk-of-bias assessment was not performed. However, the findings were interpreted considering differences in study design, including preclinical and clinical models, dosage variations, and levels of clinical validation. All figures were created using BioRender (www.biorender.com).

## 3. Results

### 3.1. Anti-Muscle-Fatigue Effects Through Natural Products

Several studies have reported that plant-based natural products, including single compounds, herbal extracts, and decoctions, exhibit anti-muscle-fatigue properties. Findings suggest that individual compounds may contribute to reducing muscle fatigue and enhancing physical performance (Table 1). Preclinical studies make up the majority of the current body of evidence and offer crucial mechanistic insights into the anti-fatigue effects of various natural products. Jiang et al. reported that polysaccharide BCP-2 extracted from *Bupleurum chinense* improved exercise endurance and reduced muscular fatigue by modulating the AMPK/PGC-1α and Nrf2/ARE pathways [33]. Tung et al. demonstrated that phenolic-rich extracts from *Calendula officinalis*, *Ribes nigrum*, and *Vaccinium myrtillus* enhanced physical endurance and muscle strength while reducing lactate and ammonia levels [34]. Hu et al. reported that curcumin from *Curcuma longa* L. extended exhaustion time, reduced lactate, and increased glycogen synthesis through the PI3K/Akt/AMPK/mTOR pathway [35]. Zhou et al. demonstrated that gastrodin supplementation reduced exercise-induced fatigue in mice by modulating the Nrf2 pathway, inhibiting inflammatory cytokines, and enhancing antioxidant enzyme activity [36]. Yang et al. identified that Ginsenoside Rg3 improved fatigue resistance in aged rats by activating SIRT1 and upregulating PGC-1α and PEPCK, highlighting its role in metabolic health [37]. Ara et al. found that hydrogen water (HW) improved swimming endurance and reduced fatigue in mice, by enhancing metabolic regulation, antioxidant capacity, and immune–redox balance [38]. Liu et al. demonstrated that lycopene supplementation improved muscle function and oxidative metabolism in high-fat-diet mice by enhancing slow-twitch muscle fibers and mitochondrial function [39]. Zheng et al. reported that macamide extracts (CME and PME) improved exercise performance in mice and reduced muscle damage through enhanced glycogen storage and reduced fatigue markers [40]. Wang et al. found that monkfish-derived peptides (LMP) enhanced endurance, reduced fatigue markers, and improved antioxidant activity in mice [41]. Li Sun et al. demonstrated that polysaccharide *Lepidium meyenii* Walp (MPS-20) increased endurance in mice and reduced fatigue markers by boosting liver glycogen content [42]. Yu et al. found that *Panax ginseng* polysaccharide (APS-1) enhanced endurance and regulated the AMPK pathway, reducing fatigue and improving glycogen levels [43]. Zhang et al. revealed that *Panax ginseng* improved chronic fatigue in rats through the PI3K/Akt/mTOR pathway, enhancing muscle endurance and recovery [44]. Hu et al. found that purple passion fruit epicarp extract (PFFA) improved endurance and glycogen storage, and reduced fatigue biomarkers [45]. Zhang et al. reported that red ginseng extracts enhanced muscle energy metabolism and mitochondrial function in chronic fatigue syndrome in mice [46]. Chen et al. found that serine supplementation reduced oxidative damage and activated Nrf2/CAR signaling to alleviate muscle oxidative stress in mice [47]. Zhang et al. demonstrated that *Hippocampus abdominalis* peptide (SH200) improved endurance, promoted mitochondrial function, and protected muscle fiber via the AMPK/PGC-1α pathway [48]. Zhang et al. reported that γ-aminobutyric acid (GABA)-enriched soymilk improved exercise performance and activated the AMPK/PGC-1α pathway, reducing fatigue markers in mice [49]. Chen et al. found that green tea catechins enhanced exercise endurance by improving ammonia detoxification and mitochondrial function in mice [50]. Ma et al. demonstrated that Chinese medicine compounds alleviated racewalking-induced fatigue in mice and improved muscle tissue integrity and waste clearance [51]. Liu et al. found that walnut oligopeptide (WOPs) increased endurance, reduced muscle damage, and enhanced recovery through improved antioxidant activity [52]. Yang et al. reported that a formula comprising wolfberry, figs, lentils, raspberries, and maca (WFWRM) improved endurance and reduced fatigue markers [53]. 

Despite the abundance of preclinical evidence, clinical trials are limited, although collectively indicating potential advantages of natural products for exercise performance, recovery, and fatigue-related outcomes. Among these, Liu et al. found that caffeinated chewing gum improved basketball performance, including shooting accuracy, sprint speed, and squat strength, while reducing fatigue in trained players [54]. Huang et al. reported that *Lactobacillus plantarum* TWK10 improved endurance, reduced lactate, and promoted better body composition [55].

Several studies have demonstrated that various herbs possess anti-muscle-fatigue effects (Table 2). Preclinical studies have extensively explored the anti-muscle-fatigue effects of various herbs using animal models. According to Egawa et al., Brazilin propolis decreased inflammatory cytokines and protected mouse skeletal muscle from glycation stress [56]. Tian et al. demonstrated that inhaling essential oils of sweet orange and lemon reduced fatigue and enhanced endurance, while bergamot aided recovery in rats [57]. Huang et al. demonstrated that *Cornu cervi pantotrichum* (CCP) improved grip strength and endurance while reducing lactate and ammonia [58]. Ho et al. found that *Coriolus versicolor* mycelia extract (CVM) improved grip strength and swimming endurance, and reduced fatigue-related biochemical markers in mice [59]. Silva de Araujo et al. reported that *Croton argyrophyllus* (HEE) extract reduced oxidative stress and muscle damage in rats after high-intensity exercise [60]. Wei et al. reported that *Dendrobium officinale* polysaccharide (DOP) improved endurance and reduced fatigue in mice with increased glycogen storage [61]. Li et al. found that *Ganoderma lucidum* and essence of chicken (CEG) improved endurance and grip strength, and reduced fatigue markers such as lactate, BUN, and CK in mice [62]. Xianchu et al. showed that grape seed proanthocyanidin extract (GSPE) extended endurance and reduced fatigue-related biomarkers while boosting antioxidant defense in mice [63]. Lee et al. showed that combining green tea extract with soy protein improved muscle strength, endurance, and glycogen stores in resistance-trained mice, enhancing recovery and performance [64]. Lu et al. demonstrated that glycoprotein from hairtail fish (*Trichiurus lepturus*) improved endurance and antioxidant enzyme activities while reducing fatigue markers in BALB/c mice [65]. Hsiao et al. observed that Hualian no.4 bitter gourd extract enhanced exercise endurance and reduced fatigue markers in mice by boosting glycogen stores and reducing lactate, ammonia, and creatine kinase [66]. 

Xia et al. concluded that processed licorice was more effective than raw licorice in enhancing glycolytic processes and metabolism for chronic fatigue syndrome (CFS) [67]. Chen et al. found that *Ludwigia octovalvis* extract (LOE) enhanced muscle glycogen storage and endurance, and reduced fatigue markers in mice, suggesting its potential as a sports supplement [68]. Saby et al. showed that melon concentrate rich in superoxide dismutase (SOD) reduced muscle damage and inflammation, improving performance and oxidative stress management in rats and humans [69]. Wang et al. detailed that *Nelumbinis stamen* reduced stress-induced muscle dysfunction and activated sestrin 2 to regulate metabolism and preserve mitochondrial function in mice [70]. Lee et al. found that *Pinus koraiensis* leaf extract (PKL) improved endurance, reduced stress markers, and enhanced tissue integrity in mice [71]. Feng et al. showed that pea peptides (*Pisum sativum* L.) increased endurance, reduced fatigue markers, and improved glycogen storage in mice [72]. Li et al. revealed that *Polygonatum cyrtonema* polysaccharides enhanced energy metabolism and ATP production via osteocalcin-mediated communication between bones and muscles, improving endurance in mice [73]. Xie Feofei et al. found that the medicinal herb *Polygala tenuifolia* alleviated fatigue by improving glycogen storage, reducing fatigue-related metabolites, and increasing antioxidant activity [74]. Yang et al. found that a traditional Chinese prescription of *Polygonati rhizoma* and *Panax notoginseng* alleviated fatigue and enhanced energy metabolism in mice [75]. Hou et al. found that *Rhodiola crenulata* oral liquid (RCOL) alleviated fatigue by enhancing antioxidant capacity and modulating mitophagy via the PINK1/Parkin pathway [76]. Dun et al. demonstrated that *Rhodiola sacra* (RS) combined with aerobic exercise enhanced mitochondrial function, reduced muscle damage, and improved endurance in mice [77]. You et al. found that *Rubus coreanus* improved swimming performance and reduced fatigue through glycogen sparing, and enhanced fat utilization and antioxidant effects [78]. Wang et al. reported that *Sarcodon imbricatus* extract boosted ATP levels, increased glycogen storage, and reduced fatigue markers through enhanced oxidative stress response [79]. Zhou et al. showed that *Schisandra chinensis* extract (SCE) extended swimming endurance and increased glycogen levels in mice [80]. Yuan et al. showed that *Sonchus arvensis* L. extract improved endurance and muscle structure while reducing fatigue-related metabolites [81]. Yin et al. reported that herbal beverage SRB improved exercise performance in mice by modulating oxidative stress and gut microbiota [82]. Yang et al. showed that *Tremella* extract enhanced endurance, boosted antioxidant activity in mice, and improved resistance to fatigue and hypoxia [83]. 

Several clinical investigations have examined the impact of herbal therapies on exercise-induced exhaustion and muscle recovery in humans. Lin et al. found that American ginseng reduced muscle damage and oxidative stress after eccentric exercise in active males via Nrf2 and AMPK pathways, lowering CK and 8-iso-prostaglandin F2α [84]. Okada et al. found that Chlorella supplementation reduced oxidative stress and improved antioxidant capacity in healthy men [85]. Loureiro et al. reported that coffee with milk enhanced muscle glycogen recovery in endurance athletes post exercise [86]. Da Silva et al. explored the impact of green tea extract supplementation on exercise-induced delayed-onset muscle soreness (DOMS) and muscular damage in untrained men [87]. Ahn et al. reported that *Gynostemma pentaphyllum* extract (GPE) improved VO_2max_ and exercise performance by modulating metabolic and antioxidant pathways [88]. Lee et al. found that Planox® lemon verbena extract (LVE) reduced muscle damage and inflammation, enhancing post-exercise recovery [29]. Buchwald-Werner et al. showed that lemon verbena extract (Recoverben®) supplementation promoted faster recovery and reduced muscle-strength loss [89]. Martin-Rincon et al. reported that mango leaf extract (Zynamite®) with quercetin reduced muscle pain and damage, accelerating recovery in humans, particularly males [90]. Shigeta et al. explored that Matcha green tea improved muscle strength and mass by modulating stress fatigue and gut microbiota composition [91]. Hunt et al. showed that New Zealand blackcurrant extract (NZBC) reduced muscle soreness and aided muscle recovery in non-resistance-trained individuals [92]. Park et al. demonstrated that *Schisandra chinensis* (SC) improved muscle strength and reduced lactate levels in postmenopausal women, highlighting its potential to reduce fatigue in adults [93]. Keller et al. examined that a higher dose of Shilajit supplementation reduced muscle-strength loss and supported connective-tissue health during fatiguing activities [94]. Hooper et al. found that tart cherry extract reduced oxidative stress and maintained muscle function after resistance exercise, aiding recovery [95]. Fernandez-Lazaro et al. found that *Tribulus terrestris* supplementation in CrossFit athletes reduced oxidative stress and enhanced recovery without significantly affecting muscle damage [96]. Soleimani et al. found that *Zingiber officinale* (ginger) supplementation attenuated exercise-induced damage and showed modest benefits for fatigue management in obese women [97].

**Figure 1:**
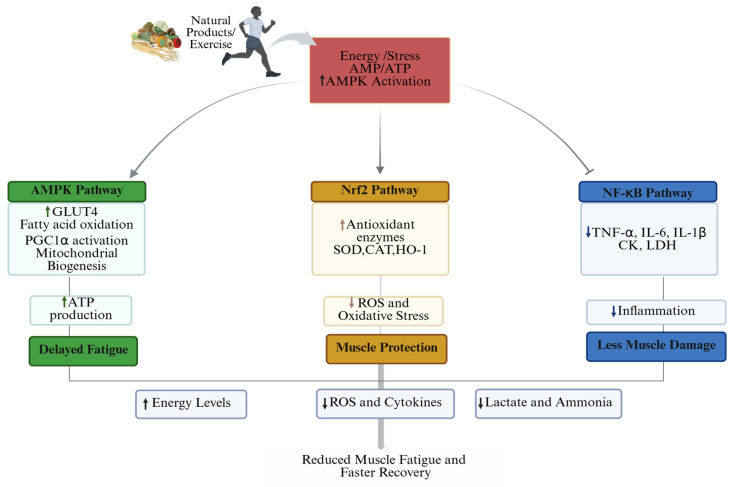
Integrated molecular mechanism of anti-fatigue effects mediated by AMPK, Nrf2 and NF-ĸB signaling pathway. Energy/stress induced elevation of AMP/ATP ratio activates AMPK, which promotes GLUT4, glucose uptake, fatty acid oxidation, mitochondrial biogenesis, and ATP synthesis to restore cellular energy homeostasis. Concurrently, Nrf2 activation upregulates cytoprotective antioxidant enzymes (SOD, CAT, HO-1) attenuating ROS-mediated oxidative damage, while inhibition of signaling suppresses pro-inflammatory cytokine production and muscle damage markers. These integrated molecular adaptations preserve metabolic efficacy, limit oxidative and inflammatory stress, and mitigate fatigue development while enhancing functional recovery. Created in BioRender. Kim, B. (2026) https://BioRender.com/huezm98 (accessed on 13 May 2026). Arrow indicates ↑ Increase/upregulation. ↓ Decrease/downregulation.

Plant-derived decoctions were found to be effective in reducing fatigue and boosting performance in various experimental studies (Table 3). Among the preclinical evidence, Wong et al. found that Huang qi polysaccharide improved grip strength and swimming endurance, and reduced lactate and urea nitrogen levels in mice [98]. Hsiao et al. demonstrated that combining *Antrodia camphorata* and *Panax ginseng* (AG) extended swimming time and grip strength, reducing lactate and CK levels in mice [99]. Miao et al. reported that Danggui Buxue Tang reduced fatigue by regulating amino acid metabolism and TCA cycle in mice [100]. Kee et al. reported that Liuwei Dihuang decoction (YJT) improved endurance and reduced fatigue in mice by modulating brain monoamines and increasing HSP70 expression [101]. Xian et al. found that *Polygonati rhizoma* polysaccharide relieved fatigue via gut microbiota modulation and enhanced energy production [102]. Zhang et al. demonstrated that Shuyu decoction improved immune function and reduced fatigue in rats, aiding exercise recovery [103]. Despite the limited number of clinical studies, Han et al. showed that Jakyakgamcho-tang improved muscle soreness and reduced biomarkers of fatigue [104]. Collectively, these findings indicate that natural products mitigate fatigue through integrated regulation of cellular energy metabolism, redox balance, and inflammatory responses. The key molecular mechanisms underlying these anti-fatigue effects are illustrated in Figure 1.

### 3.2. Enhanced Endurance from Natural Products 

The potential of plant-derived natural compounds to improve exercise endurance has been investigated in preclinical research, mostly using animal models. These studies have provided crucial mechanistic insights into their biological effects (Table 4). Chen et al. reported that arginine promoted muscle fiber type switching from fast-twitch to slow-twitch via the SIRT1/AMPK pathway, increasing the expression of slow MyHC, troponin I-SS, SIRT1, and PGC-1α, while decreasing fast MyHC [105]. Chan et al. identified that bitter melon seed oil enhanced endurance and muscle efficiency [106]. Huang et al. reported that black ginger (*Kaempferia parviflora*) improved endurance by optimizing energy metabolism [107]. Chen et al. documented that Burdock extract improved endurance and reduced fatigue markers such as lactic acid and ammonia in rats [108]. Zhong et al. observed that a *Cordyceps militaris*-based food reduced fatigue in mice [109]. Ridwan et al. found that watermelon [*Citrullus lanatus* (Thunb.) Matsum. & Nakai] improved swimming endurance and reduced lactate in rats [110]. Chang et al. reported that Danggui Buxue Tang enhanced physical performance and supported physiological adaptations during swimming exercise in rats, suggesting benefits for endurance [111].

De Andrade Soares et al. showed Acai (*Euterpe oleracea* Mart.) seed extract (ASE) supplementation improved exercise time, distance, and endurance by enhancing vascular function and mitochondrial biogenesis [112]. Su et al. reported that 2′,4′-dihydroxy-6′-methoxy-3′,5′-dimethylchalcone (DMC) enhanced endurance and fatigue resistance via the AMPK-SIRT1-PGC-1α pathway in high-fat diet-fed mice [113]. Chen et al. showed *Glossogyne tenuifolia* extract improved swimming endurance and leg strength in mice without any toxicity [114]. Lee et al. reported that *Limonium tetragonum* increased running distance, mitochondrial DNA, and oxidative fiber formation in mice [115]. Mikami et al. found that olive leaf extract improved running time and muscle weight in mice [116]. Zhang et al. reported peppermint essential oil improved exercise performance, extending exhaustion time and reducing oxidative damage in endurance-trained rats [117]. Shin et al. assessed that red ginseng enhanced endurance by promoting mitochondrial biogenesis and ATP production [118]. Liu et al. documented that *Rhodiola rosea* with caffeine boosted muscle strength and endurance [119]. Brito et al. found *Spirulina platensis* reduced exercise-induced inflammation and oxidative stress, enhancing endurance [120]. Rokkam et al. suggested that a blend of *Sphaeranthus indicus* and *Mangifera indica* improved muscle strength, muscle size, repetitions, and endurance [121]. Yada et al. found Taheebo polyphenol significantly enhanced endurance capacity by increasing muscle glycogen and reducing oxidative stress [122]. Khani et al. found thyme extract improved endurance but did not significantly affect oxidative stress or PGC-1α protein expression [123]. 

**Figure 2:**
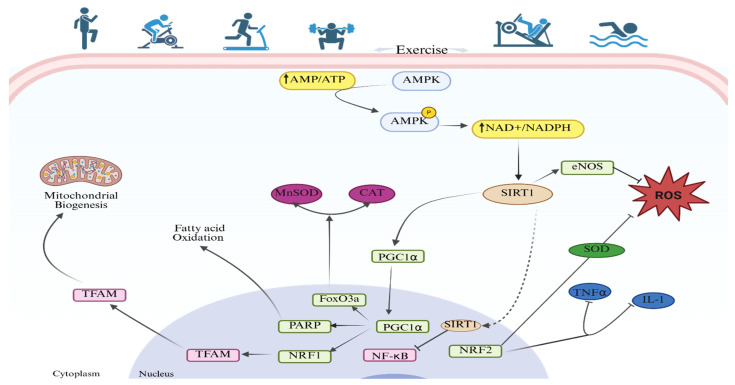
Exercise-induced activation of AMPK-SIRT1-PGC1α signaling pathways, enhances mitochondrial biogenesis, antioxidant defense, and metabolic adaptation. Activation of AMPK promotes NAD^+^/NADPH-dependent SIRT1 signaling. SIRT1 activation stimulates PGC-1α-mediated mitochondrial biogenesis and fatty acid oxidation, while also activating antioxidant defenses via Nrf2, including MnSOD and CAT. Concurrently, NF-ĸB inhibition reduces pro-inflammatory cytokines, and nitric oxide production limits ROS accumulation. Created in BioRender. Kim, B. (2026) https://BioRender.com/vzeg3qt (accessed on 13 May 2026). Arrow indicates ↑ Increase/upregulation.

Clinical studies have explored the effects of plant-derived natural products on endurance performance and exercise-related physiological outcomes (Table 4). Clinical evidence from Sousa et al. found that consuming avocado pulp (*Persea americana*) before running improved cardiovascular recovery, suggesting reduced cardiovascular risk post exercise [124]. Tan et al. demonstrated that beetroot juice during prolonged moderate-intensity exercise raised plasma nitrate and reduced oxygen uptake, although it did not affect time trial performance [125]. Kozlowska et al. reported that beetroot juice increased VO_2max_ in elite fencers [126], while Ranchal-Sanchez et al. found that muscular endurance improved during back squats [127]. Wilk et al. found that high doses of caffeine did not enhance muscle performance and caused adverse effects [128]. According to Karyigit et al., coffee consumption boosted lower-body endurance and cognitive performance without affecting heart function [129]. Sousa-Silva demonstrated that the combination of creatine with blood flow restriction training increased muscle thickness and endurance [130]. Nayyer et al. assessed that *Gynostemma pentaphyllum* improved performance and mitochondrial activity in a 20 km time trial among healthy males [131]. According to Deley et al. acute intake of grape and apple polyphenols extended maximal endurance test time and delayed exhaustion during aerobic exercise [132]. Konda et al. found that a supplement with *Garcinia mangostana* and *Cinnamomum tamala* enhanced muscle strength and endurance in resistance-trained males [133]. Sadowska-Krepa et al. determined that *Ginkgo biloba* supplementation slightly improved VO_2max_, enhancing endurance and antioxidant capacity in active males [134]. Hirsch et al. recognized that *Cordyceps militaris* supplementation enhanced high-intensity exercise tolerance, increasing VO_2max_ and the amount of time to exhaustion [135]. Anders et al. found that phosphocreatine disodium salts and blueberry extract improved peak torque and endurance performance [136]. Liao et al. revealed that *Rhodiola crenulata* and *Cordyceps sinensis* improved body composition without significantly affecting oxidative stress [137]. Collectively, these findings suggest that natural products enhance endurance through modulation of energy metabolism, mitochondrial function, and antioxidant defenses. The mechanism by which exercise-induced activation of the AMPK-SIRT1-PGC-1α pathway occurs is shown in Figure 2.

**Table 4:** Enhanced endurance from natural ingredients.

Natural Product	Single Compound/Herb/ Decoction	Animal Model/RCT(M/F)	Dose and Duration	Efficacy	Mechanism	Ref.
Preclinical Studies						
Arginine	Single compound	Male Kunming mice	0%, 0.25%, 0.5%, 1.0%/42 days	Promote transform muscle fiber type from fast-twitch to slow-twitch	↑ Slow MyHC, troponin I-SS, SIRT1, PGC-1α, SDH, MDH ↓ Fast MyHC, LDH	[105]
Bitter melon seed oil (BMSO)	Herb	Male C57BL/6Jnarl mice	15% BMSO 3 weeks	↑ Longer and further running time, muscle % ↓ Body fat	AMPK-SIRT1-PGC-1α mtDNA, Cyt c	[106]
Black ginger	Herb	Male C57BL/6J mice	62.5 mg/kg	↑ Endurance capacity	↑ Glycogen metabolism	[107]
Burdock	Herb	Male ICR mice	5 mg/kg/day/4 weeks	↑ Forelimb grip strength, exhaustive swimming time	↓ LA, NH_3_, CK	[108]
Cereal grains, *Cordyceps militaris*	Herb	Male C57BL/6J mice	5, 10, 20 g/kg body weight/30 days	↑ Swimming endurance time	↓ CK, BUN, MDA	[109]
*Citrullus lanatus* (Thunb.) Matsum. and Nakai	Herb	Male Sprague-Dawley mice	FR group, 14 days	↑ Swimming TTE	↑ NO ↓ Plasma LA, NH_3_	[110]
Danggui Buxue Tang	Decoction	Male Wistar rats	0.03, 0.18 g/mL/21 days	↑ Grip strength, endurance swimming time	↑ Muscle glycogen ↓ BLA, oxidative stress	[111]
*Euterpe oleracea* Mart.	Herb	Male Wistar Chronic rats and Acute rats	200 mg/Kg/day 5 weeks	↑ Exercise time, running distance (in chronic supplement)	↑ Nitric oxide in skeletal muscle, mitochondrial biogenesis, SOD ↓ Norepinephrine-induced vasoconstriction, MDA	[112]
2′,4′-dihydroxy-6′-methoxy-3′,5′-dimethylchalcone (DMC)	Herb	Male C57BL/6J mice	500 mg/kg; 33 days	↑ Endurance swimming, anti-fatigue	↑ ATPase, Na^+^, -K^+^AMPK-SIRT1-PGC-1α	[113]
*Garcinia mangostana* fruit rind, *Cinnamomum tamala* leaf	Herb	Swiss albino mice	CMC, 150 mg/kg GMCT, 30 mg/kg OXY/21 days	↑ Forced swimming test, forelimb grip strength	-	[133]
*Glossogyne tenuifolia*	Herb	Male ICR mice	50, 100, 250, and 500 mg/kg BW/day	↑ Forelimb grip strength, endurance swimming time	↑ Serum glucose, GOT, hepatic glycogen levels, GPT, renal markers, creatinine, uric acid levels,	[114]
*Limonium tetragonum*	Herb	Male C57BL/6J mice	30, 100 mg/kg/4 weeks	↑ Running distance (LTE 100 mg/kg)	↑ Mitochondrial DNA content, mitochondrial biosynthesis related gene expression, oxidative fiber ratio	[115]
Olive leaf extract	Herb	Male C57BL/6J mice	10 weeks	↑ Running time, EDL, gastrocnemius muscle weights ↓ Body weight, fat mass	↑ TGR5, PGC-1α, SIRT1, mitochondrial biogenesis	[116]
Peppermint	Herb	Male rats	Inhalation 4, 16, 64, 256 μL/h/2 weeks	↑ Swimming exhaustion time ↓ Body weight	↑ Serum glucose ↓ Serum LA, LDH	[117]
Red ginseng	Herb	Mice	100 mg/kg/28 days	↑ Swimming endurance	↑ Mitochondrial biogenesis regulators, NRF-1, TFAM, and PGC-1α	[118]
*Rhodiola rosea*	Herb	Male Sprague–Dawley rats	262.7 mg/kg RHO, 19.7 mg/kg CAF, 30 days	↑ 1RM, MVIC and maximal repetitions	↑ EPO, dopamine, oxygen consumption	[119]
*Spirulina platensis*	Herb	Wistar Rats	50 mg, 150 mg, 500 mg/kg/8 week	↑ Time to exhaustion	↓ CK, LDH, ROS	[120]
Taheebo polyphenol	Single compound	Male C57BL/6J mice	200 mg/kg weight	↑ Endurance capacity, running time until exhaustion	↑ Blood glucose, muscle glycogen ↓ Exercise-induced oxidative stress	[122]
Thyme extract	Herb	Male Wistar rats (20)	400 mg/kg/5 days/week for 8 weeks	↑ Endurance treadmill training	↑ MDA ↓ PGC-1α expression	[123]
Clinical Studies						
Avocado (*Persea americana*) pulp	Herb	Female (16)	600 mg, 60 min before exercise	↑ Cardiovascular, autonomic recovery after exercise	↑ Recovery of HR, systolic blood pressure, HRV, skin conductivity	[124]
Beetroot juice	Herb	Male (12)	70 mL/120 min before each visit	↑ 60%, 70% number of repetitions in back squat, 80% number of repetitions in back squat	↑ Glycogen	[125]
Beetroot juice	Herb	Elite fencers Male (10) Female (10)	26 g/day/8 weeks	↑ VO_2 max_	↑ Lipid peroxidation, β-carotene MDA, GPx, AOPP ↓ LDH, CK	[126]
Beetroot juice	Herb	Male (12)	70 mL BD, 5 weeks	↑ Oxygen uptake during exercise	↑ Plasma nitrate	[127]
Caffeine	Single compound	Male athletes (Habitual caffeine)	PLAC and 9 mg/kg/b.m+11 mg/kg/b.m /1-week interval	High doses of CAF in habitual CAF consumers may be ineffective or also have a negative effect on physical performance in athletes.	↓ PV, negative effect sizes (ES) and relative (%) ↓ T-REP, MP, PP, PV, and MV	[128]
Caffeinated coffee	Single compound	Female athletes (17)	3, 6 mg/kg/bm of caffeine/60 min prior to exercise	↑ Lower body muscular endurance, cognitive performance, upper body muscular endurance, HRV	↑ Heart rate variability, lactate, felt arousal	[129]
Creatine	Single compound	Male (17)	20 g of creatine/5 days/8 weeks	↑ Muscle thickness, muscle performance	↑ Muscle differentiation in cell cultures, satellite cell	[130]
*Gynostemma pentaphyllum*	Single compound	Male (16)	450 mg/4 weeks	↑ Swimming TTE ↓ Body weight, fat mass	↑ AMPK, mRNA ↓ Leptin	[131]
Grape and apple polyphenols	Single compound	Male (48)	500 mg/acute intake	↑ Running time ↓ Muscle pain	↑ NO production	[132]
*Garcinia mangostana* fruit rind, *Cinnamomum tamala* leaf	Herb	Young adults (38)	800 mg daily/42 days	↑ 1-RM bench press, 1-RM leg press, and leg extension repetitions	-	[133]
*Ginkgo biloba*	Herb	Male (9)	160 mg/day 6 weeks	↑ VO_2 max_	↑ Blood antioxidant capacity, SOD, CAT, GSH, BDNF	[134]
Mushroom blend containing *Cordyceps militaris*	Decoction	Male (4) Female (6)	4 g/day/ 3 weeks	↑ VT, time to exhaustion, relative peak power	↑ Oxygen delivery, angiogenesis, glucose uptake	[135]
Phosphocreatine disodium salts plus blueberry extract	Herb	Male (11)	5 g PDS, 200 mg blueberry/day 4 weeks	↑ MID, peak torque, average power	↑ Creatine absorption	[136]
*Rhodiola*/*Cordyceps* based herbal supplement	Herb	Male (8) Female (6)	20 mg/kg/day/5 days/8 weeks	↑ Endurance training	-	[137]

Arrow indicates ↑ Increase/upregulation. ↓ Decrease/downregulation.

### 3.3. Enhancement of Muscular Strength by Natural Products

Preclinical research has widely explored plant-derived natural products for their effects on skeletal muscle performance, demonstrating effects on muscle strength, hypertrophy, regeneration, and protection against muscle damage across various experimental models. Based on available evidence, Yeh et al. reported that *Astragalus membranaceus* promoted myotube hypertrophy via the PI3K/Akt/mTOR pathway, increasing myotube diameter and muscle hypertrophy, which were reduced with PI3K or mTOR inhibitors [138]. Shi et al. found that *Achyranthes bidentata* saponin extract (ABSE) mitigated muscle atrophy via the PI3K/Akt signaling pathway, preserving muscle mass [139]. Cheng et al. demonstrated that *Cordycepin* inhibited muscle differentiation by activating the ERK1/2 MAPK pathway, reducing differentiation markers, preserving undifferentiated cells, and decreasing oxidative stress [140]. Lin et al. documented that Epimedium extract and icariin promoted skeletal muscle cell hypertrophy by activating IGF-1/PI3K/Akt signaling and related pathways [141]. Jatwani and Tulsawani demonstrated that *Ganoderma lucidum* reduced muscle oxidative stress and aided muscle regeneration under hypoxic conditions [142]. Jiang et al. found that grilled nux vomica (GNV) ameliorated autoimmune myasthenia gravis symptoms by inhibiting the TLR4/NF-κB pathway [143].

Zhu et al. found that the medicinal herb *Lepidium meyenii* (Maca) enhanced muscle strength and endurance while alleviating exercise-induced fatigue through improved energy metabolism and antioxidant capacity [144]. Jeong et al. found that Korean mistletoe extract (KME) regulated muscle growth and atrophy by activating the PI3K/Akt pathway and suppressing the atrophy genes (MuRF1), leading to increased muscle mass and grip strength [145]. Han et al. reported that *Panax ginseng* berry extract and whey protein hydrolysate improved muscle strength and prevented atrophy in aged mice by increasing grip strength and muscle mass, and reducing protein degradation and inflammation [146]. Roumanille et al. demonstrated that *Rhaponticum carthamoides* and *Rhodiola rosea* extracts enhanced muscle strength and recovery after resistance exercise [147]. 

Qi et al. demonstrated that vine tea extract (VTE) alleviated fatigue by enhancing muscle mass and energy metabolism through AMPK/FOXO signaling pathways, increasing swimming time, elevating glycogen levels, reducing lactic acid accumulation, and downregulating the atrophy-related gene FOXO1 in older adults [148]. Panda et al. found that *Withania somnifera* extract boosted muscle strength and reduced oxidative stress in aging rats [149].

**Figure 3:**
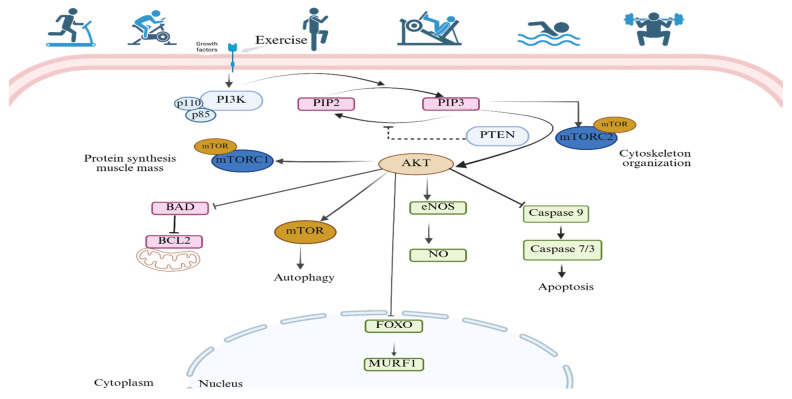
Mechanistic overview of PI3k-Akt-mTOR signaling pathways in exercise-induced cellular adaptations. Exercise and growth factors activate PI3K, promoting PIP2-to-PIP3, conversion, and Akt activation, which is negatively regulated by PTEN. Activated Akt stimulates mTORC1 and mTORC2 to enhance protein synthesis, activates eNOS to increase NO production, inhibits apoptosis via BAD and caspase 9/3, and suppresses FOXO-MURF1-mediated protein degradation, orchestrating multiple downstream effects. This coordinated effect promotes muscle hypertrophy, survival, and metabolic adaptation. Created in BioRender. Kim, B. (2026) https://BioRender.com/we05poj (accessed on 13 May 2026).

Clinical investigations have further explored the translational potential of plant-derived natural products in enhancing muscle strength and exercise performance (Table 5). Among these studies, Cook et al. found that New Zealand blackcurrant extract (NZBC) lowered systolic blood pressure post-exercise, increased fat oxidation, and improved metabolic efficiency during moderate-intensity exercise by promoting vasodilation through nitric oxide pathways [150]. Chen et al. demonstrated that caffeine supplementation via caffeinated gum improved performance in Roman deadlifts, increasing peak and average power, while heart rate and perceived exertion remained unchanged [151]. Pirmohammadi et al. reported that early caffeine absorption strategies, including chewing caffeinated gum and coffee mouth-rinsing, enhanced performance in female table tennis players, particularly in motor function and cognition [152]. According to Tripathi et al. *Chlorophytum borivilianum* (CB) improved physical performance, cardiovascular function, and muscle strength, showing adaptogenic and anti-stress properties [153]. Van Iersel et al. found that citrus flavonoid extract (CFE) enhanced anaerobic capacity, increasing power output and performance during high-intensity exercise after 4 weeks of 400 mg/day supplementation, improving energy metabolism and reducing oxidative stress [154]. Tokuda et al. found that essential amino acids (EAA) and tea catechins (TCC) improved skeletal muscle mass in older adults with sarcopenia after resistance exercise, highlighting the role of nutritional intervention in preventing muscle loss [155]. Oxfeldt et al. reported that fermented red clover extract helped preserve muscle by downregulating protein degradation markers (FOXO1, FOXO3a) and increasing HSP27 in postmenopausal women, suggesting potential to reduce muscle atrophy and promote protein synthesis [156]. Gelabert-Rebato et al. reported that mango leaf extract (Zynamite®) with quercetin enhanced peak power output during sprint and improved metabolic efficiency [157]. According to Priya et al. a synergistic marmalade formulation made from purple grapes, watermelon, and beetroot improved muscle performance and ergogenic activity in athletes by improving phosphocreatine resynthesis, muscle power, and relieving soreness and fatigue [158]. William et al. found that *Rhodiola rosea* (Golden Root) improved explosive performance by increasing norepinephrine, although it reduced endurance capacity during resistance exercise [159]. Perez-Pinero et al. reported that spinach extract boosted muscle strength and quality in adults over 50 years of age by promoting protein synthesis; 12 weeks of supplementation improved knee extension torque and reduced fat mass [160]. Kang et al. reported that a 12-week regimen of whey protein supplementation alongside resistance exercise improved muscle function in older adults [161]. Ziegenfuss et al. demonstrated that *Withania somnifera* (Ashwagandha) supplementation (500 mg/day) enhanced strength and recovery during resistance training, improving performance in exercises such as bench press and squat [162]. Figure 3 illustrates that exercise modalities, including endurance, resistance, swimming, and cycling modulate the PI3K/Akt pathways, promoting muscle growth, survival, and metabolic adaptation.

### 3.4. Activation of Muscular Nervous System from Natural Products

**Figure 4:**
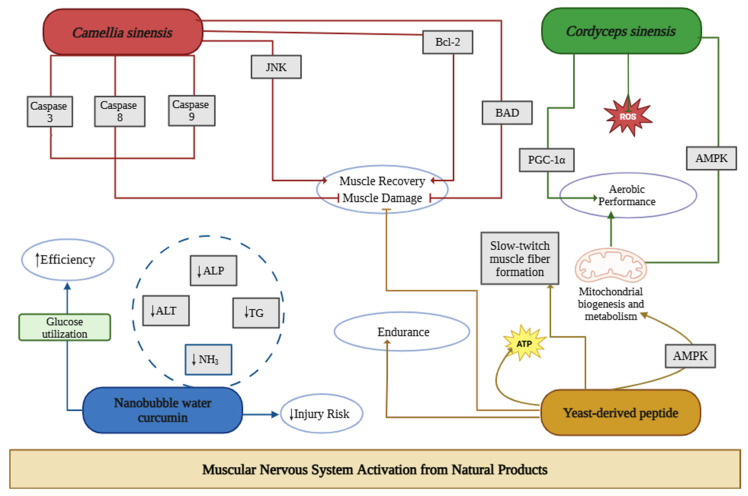
The pathways involved in neuromuscular system activation by natural products. Created in BioRender. Kim, B. (2026) https://BioRender.com/p7kwg9h (accessed on 13 May 2026). Arrow indicates ↑ Increase/upregulation. ↓ Decrease/downregulation.

Four studies have explored the potential health benefits of polyphenols, *Cordyceps sinensis*, nanobubble curcumin extract, and yeast peptide (Figure 4 and Table 6). A preclinical study supported these effects mechanistically, as Cai et al. reported that a yeast-derived peptide (YPLP) enhanced muscle function by activating the AMPK/PGC-1α pathway, improving mitochondrial function, promoting slow-twitch fiber formation, and reducing fatigue markers, thereby enhancing endurance and recovery in mice [163].

A few clinical studies further suggest translational benefits in performance and recovery. Extending these findings, Townsend et al. reported that 28 days of polyphenol supplementation from *Camellia sinensis* (tea) reduced muscle cell apoptosis after resistance exercise by lowering apoptotic c-Jun N-terminal kinase (JNK) and Bcl-2-associated death promoter (BAD) phosphorylation, aiding recovery by limiting cellular damage [164]. Savioli et al. found that *Cordyceps sinensis* supplementation boosted aerobic performance in marathoners, reducing heart rates during submaximal exercise and improving 5 km run times, suggesting enhanced endurance and cardiovascular efficiency [165]. Wang et al. demonstrated that nanobubble curcumin extract (NCE) reduced injury risk and muscle damage markers (alanine transaminase, triglycerides, and lactate) and improved recovery by boosting high-density lipoprotein (HDL) and glucose utilization [166].

### 3.5. Anti-Inflammatory Effects of Natural Products on Exercise-Associated Inflammation

**Figure 5:**
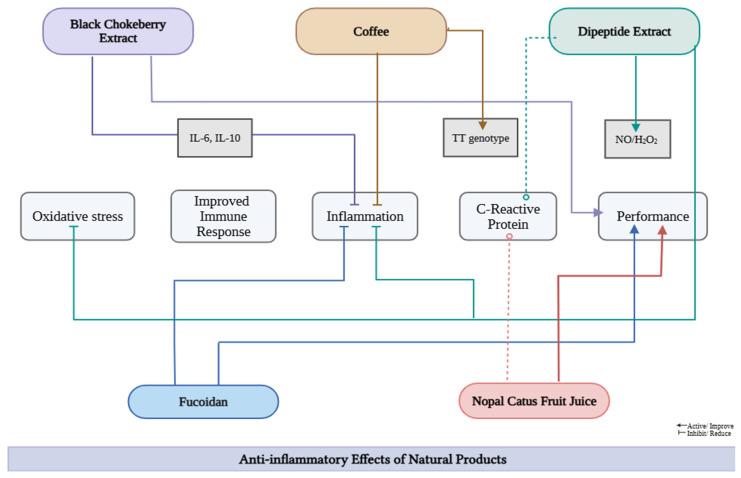
Schematic representation of anti-inflammatory mechanisms of natural products in exercise-induced inflammation. Created in BioRender. Kim, B. (2026) https://BioRender.com/eys0o8y (accessed on 13 May 2026).

Clinical investigations have revealed that some plant-based compounds/extracts can reduce inflammation (Figure 5 and Table 7). Stankiewicz et al. reported that black chokeberry (*Aronia melanocarpa*) extract significantly improved inflammatory markers and antioxidant status, enhancing performance, and reducing inflammation in athletes [167]. Rahimi et al. found that individuals with the adenosine A2A receptor (ADORA2A) TT genotype experienced reduced inflammation from caffeine intake after resistance exercise [168]. Zembron-Lacny et al. demonstrated that a dipeptide extract reduced oxidative stress and improved the antioxidant defense system during high-intensity endurance exercise [169]. According to McFadden et al., fucoidan supplementation significantly improved inflammatory and immune responses after high-intensity exercise [170]. Jensen et al. found that nopal cactus fruit juice consumption enhanced joint mobility and endurance by reducing inflammation in a placebo-controlled trial [171].

### 3.6. Other Benefits 

There have been published studies reporting additional efficacies of natural products beyond those of the aforementioned outcomes (Table 8). The available evidence comprises both clinical and preclinical studies, with preclinical investigations providing mechanistic insights and human studies offering preliminary evidence of the efficacy of natural products in exercise performance. Among preclinical studies, Kweon et al. found that *Angelica keiskei* ethanol extract (EAK) protected against dexamethasone-induced muscle atrophy, and its active compound 4-hydroxyderricin enhanced myogenesis by activating the p38 MAPK pathway, suggesting potential therapeutic applications [172]. Daussin et al. reported that cocoa flavanol (CF) supplementation improved mitochondrial function, NAD^+^ and NADH levels, and carbohydrate utilization in mice, with these effects attenuated in SIRT3 knockout mice [173]. Tayebi et al. demonstrated that cinnamon supplementation during swimming training reduced haemoglobin A1c (HbA1c) and the expression of TBC1D1 and TBC1D4 proteins in diabetic rats, improving glucose management and insulin sensitivity [174]. Tian et al. reported that low doses of green tea catechins, including epigallocatechin gallate and epicatechin gallate, extended lifespan and improved stress resistance in *Caenorhabditis elegans* by inhibiting mitochondrial complex I and enhancing antioxidant defense via AMPK, SIRT1, and p38 MAPK pathways [175]. Ruiz-Iglesias et al. reported that hesperidin supplementation enhanced intestinal IgA synthesis in rats under chronic physical stress and strengthened the mucosal barrier [176]. Ishida et al. found that Juzentaihoto (JTT) improved muscle function and insulin resistance in a mouse model of obesity and type 2 diabetes [177]. Yamamoto et al. reported that Lemon Myrtle (*Backhousia citridora*) extract and its active compound casuarinin enhanced satellite cell activation and muscle regeneration in vitro and in vivo [178]. Kim et al. reported that *Salvia plebeia* and rosmarinic acid attenuated dexamethasone-induced muscle atrophy in C2C12 myotubes by improving cell viability and mitochondrial function via the Akt/mTOR pathway [179]. O’Leary et al. reported that Shatavari supplementation did not significantly alter skeletal muscle proteome or training adaptation pathways in postmenopausal women [180]. Zhao et al. demonstrated that Xiaoyao San (XYS) improved exercise capacity and depressive symptoms in rats by regulating liver mitochondrial metabolomics and enhancing energy metabolism [181]. Wu et al. reported that Yiqi Chutan formula (YCF) alleviated cisplatin-induced muscle damage by reducing oxidative stress and ferroptosis, thereby protecting muscle cells [182].

Among clinical studies, Chung et al. reported that Aronia berry extract (ABE) strengthened the glutathione defense system in middle-aged adults, particularly countering oxidative stress after aerobic exercise by increasing glutathione (GSH) availability and glutathione peroxidase (GPx) activity [183]. Ismansyah et al. demonstrated that bay leaf decoction combined with low-impact exercise reduced blood cholesterol in individuals with hypercholesterolemia [184]. Moss et al. reported that a single dose of New Zealand blackcurrant (NZBC) extract improved 5 km run times in trained male runners without altering physiological or metabolic responses during exercise and enhanced muscle oxygen delivery [185]. Kanazashi et al. found that Brazilian propolis reduced body fat and oxidative stress in elderly females, mitigating obesity-related metabolic disturbances [186]. Delfan et al. reported that broccoli sprout supplementation, particularly when combined with exercise training, improved apolipoprotein profiles, glycemic control, and insulin sensitivity in men with T2DM, although the benefits were primarily driven by exercise [187]. Flockhart et al. reported that supplementation with glucosinolate-rich broccoli sprout (GRS) enhanced physical performance and attenuated oxidative stress and inflammation by decreasing skeletal muscle protein carbonylation and plasma myeloperoxidase levels via activation of antioxidant defense pathways [188].

Kaszuba et al. reported that caffeinated chewing gum improved attack accuracy in volleyball players without affecting other physical performance metrics [189]. Trujillo-Colmena et al. demonstrated that soluble coffee containing 3 mg/kg of caffeine improved cross-country cycling performance, reducing trial time by 4.93% without increasing perceived exertion [190]. Jyvakorpi et al. found that coffee consumption was positively correlated with improved physical performance, including gait speed and chair rise in elderly men [191]. Ghaesmi et al. found that combining high-intensity interval training (HIIT) with green tea supplementation improved metabolic and antioxidant responses in overweight women and increased SIRT1, PGC-1α, and catalase, enhancing antioxidant capacity, body composition, and aerobic performance [192]. Pahlavani et al. demonstrated that L-arginine supplementation improved performance in male soccer players without affecting body composition [193]. Przewlocka et al. found that combined probiotic and vitamin D3 supplementation improved anaerobic performance in male mixed martial arts (MMA) athletes [194]. Owen et al. found that a high-dose of vitamin D3 supplementation in elite athletes elevated serum vitamin D concentrations, suggesting that frequent dosing may be more effective and safer for long-term health [195].

## 4. Discussion

### 4.1. Mechanistic Insights of Natural Products

Natural product interventions have been reported to be associated with reductions in muscle fatigue and oxidative stress, alongside improvements in endurance and muscle strength. Most studies reviewed here were multi-compositional with varied study designs, dosages, intervention durations, and experimental models. Several isolated bioactive compounds appear to modulate fatigue-related pathways, particularly those involved in energy metabolism and oxidative stress regulation. The AMPK/PGC-1α pathway, which is critical for mitochondrial biogenesis and energy homeostasis, is modulated by key compounds such as *Bupleurum chinense* polysaccharide, ginsenoside Rg3, GABA-enriched soya milk, lycopene, and *Hippocampus abdominalis* peptide SH200. These agents have been reported to influence ATP production, oxidative metabolism, and fatigue resistance, in line with the findings reported by Rao et al. that AMPK/PGC-1α activation enhances mitochondrial function and reduces oxidative stress and inflammation, leading to improved exercise performance and fatigue resistance [196]. Similarly, the Nrf2 pathway, a key regulator of antioxidant defense, is modulated by compounds like gastrodin, serine, monkfish peptide, and walnuts, which enhance antioxidant capacity, consistent with the findings by Kim et al. who demonstrated performance improvement through Nrf2 and PGC-1α activation [197]. 

Bioactive extracts from natural products have been reported to modulate pathways involved in energy utilization (PGC-1α, PPARα, mTOR) [198], glycogen synthesis (PI3K/Akt) [31], oxidative stress regulation (Nrf2/ARE), mitochondrial autophagy (PINK1/Parkin) [32], and muscle protein synthesis, hypertrophy, and inflammation regulation (NF-κB, MAPK, ERK) [199]. Compounds such as curcumin, *Panax ginseng*, and red ginseng activate the PI3K/Akt/mTOR pathway to enhance glycogen synthesis, muscle repair, and endurance, consistent with evidence from Lee et al. who reported that PI3K/Akt/mTOR reactivation promotes muscle protein synthesis and attenuates muscle atrophy by regulating the ubiquitin-proteasome system [200]. Collectively, these findings provide a biologically plausible mechanistic rationale for the anti-fatigue and endurance-enhancing effects of natural products. However, human evidence supporting these mechanistic findings remains sparse.

### 4.2. Functional Performance and Recovery

The intensity, duration, and type of exercise are critical factors influencing physiological responses and recovery [201,202]. Garcia et al. emphasized that post-exercise recovery is essential for injury prevention, fatigue management, and sustained performance [203]. Recovery strategies have been categorized into passive (massage, nutrition), active (low-intensity exercise), and proactive approaches (mindfulness and social support) [204].

Several herbs included in this study have demonstrated potential anti-fatigue effects, associated with enhanced antioxidant defense and reduced muscle damage biomarkers such as creatine kinase (CK), malondialdehyde (MDA) and pro-inflammatory cytokines. These effects contribute to improved endurance and recovery, with reported effective doses ranging from 300–500 mg/kg in animal models and approximately 400–1000 mg/day in human studies. Prior research has identified oxidative stress as a major contributor to muscle fatigue [205]. Recent work by Irawan et al. further supports this concept, showing that bioactive compounds protect against exercise-induced oxidative stress and inflammation, attenuate post-exercise IL-6 and CK responses, and demonstrate practical feasibility as recovery-promoting interventions [206]. 

Our present study identified plant-based decoctions such as *Antrodia camphorata*, *Panax ginseng*, Dannui Buxue Tang, and Shuyu decoction as promising interventions for reducing lactate, regulating the TCA cycle and amino acid metabolism, and modulating gut microbiota and immune function. These findings underline the potential of natural products for improving functional performance and recovery. In parallel, Pico et al. reported that traditional herbal preparations combined with adjunctive practices including massage, acupuncture, and moxibustion contribute to meaningful alleviation of fatigue and emotional distress, while supporting quality of life without compromising safety [207]. Although anti-inflammatory effects have been reported for black choke berry, caffeine, dipeptide extract, nopal cactus juice, and fucoidan, these responses appear to be genotype-specific during high-intensity endurance exercise. This variability limits the generalizability of the findings, making it necessary to determine which populations benefit most and under what training conditions.

### 4.3. Synergistic and Holistic Benefits

Various plant-based compounds, despite differing origins, activate complementary biological pathways that collectively promote muscle regeneration, metabolic adaptation, antioxidant defense, and exercise recovery. Aronia berry extract, Lemon, *Achyranthes bidentata*, saponin extract, and *Angelica keiskei* engage in pathways such as PI3K/Akt and p38 MAPK, supporting muscle recovery and function [208,209]. Green tea catechins and cocoa flavanols activate the AMPK pathway, enhancing mitochondrial function and antioxidant defense [210,211]. These findings are consistent with previous studies emphasizing mitochondrial biogenesis and improved fat oxidation [212,213]. The commonality in these molecular pathways suggests potential synergistic effects, a hypothesis supported by studies reporting the benefits of multi-compound supplementation [214]. However, systematic evidence for synergistic effects in humans remains limited, and potential antagonistic interactions, optimal compound combinations, dosage strategies, and long-term safety of multi-compound regimens remain largely unexplored [215].

### 4.4. Targeted Population and Safety Considerations

The effects of natural product supplementation appear to be context-dependent and should be interpreted in relation to population-specific physiological demands and training status [216]. Nutritional and exercise interventions in older adults indicate that specific supplements may contribute to improvements in physical performance, muscle function, and exercise tolerance. However, the magnitude of these effects varies depending on sex, baseline fitness status, and type and dosage of supplementation. Therefore, individualized nutritional strategies are recommended to optimize functional outcomes in this population [217]. Women mainly use supplements for health maintenance, energy enhancement, and correcting dietary inadequacies [218]. Among athletes, natural product supplementation may support athletic performance, accelerate recovery, and mitigate exercise-induced physical stress, particularly when dietary intake is insufficient to meet the higher metabolic and training demands [219]. Similarly, in physically active men, these interventions enhance performance outcomes such as repetitions to failure, power output, and fatigue resistance [220]. 

Although natural products have demonstrated potential benefits for exercise performance and recovery, their safety should be considered alongside their efficacy [221]. While intervention studies have generally reported good tolerability, evidence on long-term safety of chronic or high-dose supplementation remains limited. Compared with dietary sources, concentrated supplement formulations may substantially increase bioactive exposure, potentially increasing the risk of adverse effects, herb-drug and nutrient interactions, contamination, adulteration, and variability in bioavailability and product quality [222]. Analytical studies have reported that 14–15% of sports supplements contain undeclared substances, underscoring the importance of third-party quality certification and careful product selection [223]. Excessive intake of antioxidant-rich supplements may impair beneficial exercise-induced adaptations, including mitochondrial biogenesis, endogenous antioxidant defense, and skeletal muscle adaptations, as demonstrated by high-dose vitamin C and vitamin E supplementation in both young and older adults, although short-term exercise performance outcomes remain unaffected [224,225]. Therefore, natural product supplementation should be personalized according to dosage, training/activity status, and physiological conditions for long-term safety. 

### 4.5. Implications and Translational Relevance

The findings of this review have important implications for research and practice in sports medicine, exercise physiology, and public health. In this context, current evidence suggests that plant-based compounds and natural extracts may serve as promising adjuncts to conventional training, with potential benefits for endurance, muscle strength, neuromuscular activation, and recovery. Collectively, these findings support their use as complementary interventions within structured training and nutritional programs. However, individual characteristics, including age, sex, genetic variability, and training status should be considered when developing personalized supplementation strategies to maximize effectiveness. Furthermore, these insights may facilitate the development of functional foods, nutraceuticals, and sport-specific supplements designed to support performance, recovery, and injury prevention. Moreover, interdisciplinary research combining exercise physiology, molecular biology, and nutrition science is encouraged to investigate multi-compound synergy, establish optimal dosing, and confirm long-term safety in diverse populations. 

The translational potential of natural products in exercise remains promising but has not yet been fully established in human populations [226]. Current evidence is largely derived from preclinical studies and small-scale or heterogeneous clinical trials, which limit the strength of real-world applicability. However, the difference between animal and human studies should be interpreted cautiously in translational research, as dose equivalence, metabolic processes, and exercise protocols vary substantially across species [227]. The gap between experimental findings and standardized clinical application underscores the need for rigorously designed, well-powered human trials with harmonized protocols. Accordingly, natural product interventions should currently be considered promising complementary strategies, although further clinical validation is required before they can be established as evidence-based ergogenic approaches.

### 4.6. Limitations

Despite the comprehensive coverage of natural products in exercise performance and recovery, several limitations merit critical consideration. The available evidence may also be affected by publication bias, as studies reporting beneficial effects are more likely to be published than those with null or inconclusive findings. A substantial proportion of the available evidence is derived from preclinical models, which provide valuable mechanistic insights but require further validation in human physiology. Moreover, most studies focus on short-term outcomes, including endurance, fatigue reduction, oxidative stress markers, and exercise performance, while adverse events and long-term safety were inconsistently reported. Consequently, firm conclusions regarding the safety, sustainability, and efficacy of natural product supplementation cannot yet be drawn. Potential issues related to supplement quality, contamination, or adulteration were also rarely assessed in the included studies. Furthermore, the magnitude of reported benefits may vary according to the intervention, dosage, participant characteristics, and exercise conditions, indicating that responses may not be uniform across all populations. Variability in study populations with respect to age, gender, fitness level, and health status further restricts the generalizability of the findings. Although some investigations elucidate underlying molecular mechanisms, many studies lack mechanistic validation in humans, leaving gaps in understanding the precise biological pathways involved. 

### 4.7. Future Prospects

Advancing natural products for performance enhancement will require standardized extraction, robust dose–response profiling, and long-term clinical trials coupled with mechanistic validation of underlying pathways. Artificial intelligence-based approaches, including machine learning, network pharmacology, and multi-omics integration, may accelerate target identification, predict synergistic interactions, and optimize compound selection. Combining genetic and physiological profiling with AI modeling could enhance precision-oriented supplementation strategies. Future research should also prioritize standardized intervention protocols, multicenter clinical validation, and regulatory frameworks to facilitate the safe and effective translation of promising natural products into evidence-based practice.

## 5. Conclusions

Natural products represent a promising and rapidly expanding area of research in exercise performance and recovery. The evidence reviewed indicates that a wide range of bioactive compounds and natural extracts can modulate key physiological processes involved in fatigue resistance, energy metabolism, oxidative stress regulation, inflammation control, and muscle adaptation. These effects have been associated with improvements in endurance capacity, muscle function, recovery, and overall exercise outcomes across diverse experimental and clinical settings. Although the evidence is promising, the quality of evidence varies among interventions, with some natural products supported by human studies and others requiring further clinical validation. Nevertheless, natural products are biologically plausible and potentially useful as complementary strategies for exercise performance and recovery; however, they are not yet sufficiently validated to support broad performance recommendations without stronger evidence from rigorous human trials. Future well-designed clinical studies will be important for establishing optimal dosing, confirming long-term safety and efficacy, and facilitating evidence-based applications across populations with varying levels of physical activity and age.

## Figures and Tables

**Table 1:** Anti-muscle fatigue effect by single compound.

Natural Product	Animal Model/RCT(M/F)	Dose and Duration	Efficacy	Mechanism	Ref.
Preclinical Studies					
BCP-2	Male ICR mice	200 mg/kg; 30 days	↑ Forced swimming time	↑ Serum LG, MG, SOD, GSH-Px, Nrf2 ↓ LA, BUN, LDH, CK, MDA, Keap1	[33]
*Calendula officinalis*, *Ribes nigrum*, *Vaccinium myrtillus*	Male ICR mice	80, 160, 400 mg/kg; 5 weeks	↑ Forelimb grip strength, ↑ Exhaustive swimming time	↑ Muscle glycogen ↓ LA, NH_3_, glucose, BUN, CK	[34]
Curcumin	Male Kunming mice	250 mg/day; 28 days	↑ Treadmill exhaustive time	↑ Quadriceps coefficient, GS, myonectin, MG, mTOR ↓ AMP/ATP, PI3K, Akt, AMPK	[35]
Gastrodin	Male C57BL/6 mice	200 mg/kg/day; 30 days	↑ Average velocity, number of activities, activity time, degree of linearity, forelimb grip strength, swimming time ↓ Rest time	↑ LG, MG, SOD, GSH-Px, Nrf2, HO-1, NQO1 ↓ BLA, LDH, MDA, IL-1β, TNF-α, IL-6, Keap1	[36]
Ginsenoside Rg3	Male Sprague-Dawley rats	20 mg/kg; 10 days	↑ Journey, rearing time ↓ Resting time	↑ SOD, PGC-1α, PEPCK, SIRT1 ↓ MDA, LDH, TG	[37]
Hydrogen Water	Female C57BL/6 mice	1.0–1.2 ppm; 4 weeks	↑ Swimming endurance time	↑ Liver glycogen, GPx, catalase, Serum LDH ↓ Blood glucose, LA, BUN, serum NO, IL-1β, IL-6, IL-17, TNF-α	[38]
Lycopene	Male C57BL/6 mice	0.33% lycopene; 8 weeks	-	↑ Slow-Twitch Fiber (MyHCI, TNNC1, MEF2C, PGC-1α), MDH, SDH, fatty acid synthesis (SREBP1C, DGAT1, DGAT2, C/EBP α, ACC α), mitochondrial ATP production ↓ Fast Twitch Fiber (MyHCIIb, MYH4, TNNC2), LDH, PK, lipolysis-related genes (ATGL, PPAR γ), MDA	[39]
	C2C12 cells	10 μM; 24 h	↑ Cell proliferation, myotube differentiation	↑ Mitochondrial ATP production ↓ Glycolytic reserve	
Macamide	Male Kunming mice	CME: 30, 120 mg/kg PME: 8, 32 mg/kg; 28 days	↑ Exhaustive swimming time	↑ MG, LG ↓ BUN, BLA, LDH, CK, free fatty acid, skeletal muscle damage, myocardium	[40]
Monkfish (MPH)	Male Kunming mice	333, 667, 1000 mg/kg; 4 weeks	↑ Exhaustive swimming time	↑ HG, SOD, GSH-Px, CAT, NK ↓ BUN, BLA, MDA, LDH	[41]
MPS-1, MPS-2	Male Kunming mice	20, 100 mg/kg; 30 days	↑ Forced swimming time	↑ LG ↓ BLA, BUN, LDH	[42]
*Panax ginseng* C. A. Meyer (APS-1)	Male C57BL/6J mice	50, 100, 150 mg/kg; 15 days	↑ Exhaustive swimming time	↑ SOD, CAT, LKB1, p-AMPK, PGC-1 α, GLUT4 ↓ BLA, LDH, BUN, MDA	[43]
*Panax ginseng* C. A. Meyer (*Araliaceae*)	Sprague-Dawley rats	50, 100 and 200 mg/kg; 15 days	↑ Physical recovery, energy utilization, PI3K/Akt/mTOR	↑ Swimming time, glycogen, hepatic glycogen, blood glucose, ATPase activities ↓ Serum triglycerides, total cholesterol	[44]
PFEA	Female ICR mice	200, 400, 600 mg/kg; 30 days	↑ Forced swimming time	↑ LG, SOD, PPARα, PGC-1α ↓ BLA, BUN, LDH, MDA, TNF-α, IL-6, IL-1β	[45]
Red ginseng extract	C57BL/6J mice	200, 400, 600 mg/kg; 28 days	↑ Loaded swimming time, rotarod time	↑ p-AMPK, PGC-1α, Na^+^-K^+^-ATPase, CcO, ATP, complex I ↓ LA, LDH, urea	[46]
Serine	Male C57BL/6J mice	1.5 g/kg BW/day; 4 weeks	↑ Running time, distance, exercise performance	↑ Citrate synthase, CAT, CuZnSOD, MnSOD, GSH, GSH/GSSG, GPX, GRD, GST, nuclear Nrf2, nuclear CAR ↓ ROS, hydrogen peroxide, superoxide radical, 8-OHdG protein carbonyl, MDA, 8-isoprostane, complex I	[47]
SH200	C57BL/6 mice	200 mg/kg; 4 weeks	↑ Rota-rod time	↑ Hepatic glycogen, ATP, SDH, MYH, PGC-1α ↓ LA, LDH, CK	[48]
Soymilk	Male C57BL/6 mice	4 weeks	↑ Forced swimming time	↑ LG, MG, muscle ATP, SOD, GSH-Px, PPARα ↓ BLA, BUN, LDH, MDA	[49]
Green tea catechins	Male BALB/c mice	0.2 g/kg; 4 weeks, 0.5% GTC diet; 8 weeks	↑ Exercise endurance	↑ Hepatic Nags, Cps1, Asl, Arg1 ↓ Blood NH_3_	[50]
	C2C12 myoblasts	0.001, 0.01% GTC; 24 h	-	↑ OCR	
Traditional Chinese medicine	ICR mice	**-**	-	↑ Nrf2, Bcl-2 ↓ BUN, LA, LDH, CK	[51]
Walnut (WOP)	Male ICR mice	110, 220, 440 mg/kg; 30 days	↑ Swimming time	↑ mtDNA, LG, gastrocnemius glycogen, SDH, SOD, GPX, Na^+^-K^+^-ATPase ↓ LDH, CK, BUN, BLA, MDA	[52]
WFWRM	Male Kunming mice	1.00 g/kg; 30 days	↑ Rota-rod time	↑ LG, MG, GLU, SOD, GSH-Px ↓ LA, BUN, LDH, CK, MDA	[53]
Clinical Studies					
Caffeinated gum	Adult male basketball players (15)	3 mg/kg; 10 min	↑ Free throw accuracy Sprint test, running anaerobic sprint test	↑ Peak concentric and eccentric power, minimum power ↓ Fatigue index	[54]
TWK10	Healthy adults (54)	1 × 10^10^, 3 × 10^10^ CFU/day; 6 weeks	↑ Exhaustion time	↑ Glucose, muscle mass ↓ LA, NH_3_, fat mass	[55]

Arrow indicates ↑ Increase/upregulation. ↓ Decrease/downregulation.

**Table 2:** Anti-muscle fatigue effect by herbs.

Natural Product	Animal Model/RCT(M/F)	Dose and Duration	Efficacy	Mechanism	Ref.
Preclinical Studies					
Brazilian Propolis	Male C57BL/6NCr mice	0.1% of diet; 20 weeks	↓ AGEs accumulation ↓ Inflammation markers, muscle-mass protection	↑ Glyoxalase 1 activity, MGO-derived AGE detoxification ↓ IL-1β, IL-6	[56]
Citrus Essential Oils (SEO, LEO, BEO)	Male Sprague Dawley rats	1 mL/cage; 36 days	↑ Swimming time	LEO: ↓ Glucose, MDA SEO: ↓ BLA, ↑SOD BEO: ↑ GSH-Px ↓ BLA, MDA, SOD CEO: ↓ LG, CK, MG	[57]
*Cornu cervi pantotrichum*	Male ICR mice	4108 mg/kg; 6 weeks	↑ Forelimb grip strength, Endurance swimming time	↑ BAT ↓ NH_3_, CK	[58]
*Coriolus versicolor* Mycelia Extract	Male ICR mice	615, 1230, 3075 mg/kg/day; 4 weeks	↑ Forelimb grip strength ↑ Exhaustive swimming time	↓ LA, NH_3_, CK ↓ Glucose, TG	[59]
*Croton argyrophyllus*	Female rat	200 mg/kg	-	↓ CK, LDH, MDA	[60]
*Dendrobium officinale*	Male BALB/c mice	50 mg/kg; 30 days	↑ Swimming time, food consumption rate	↑ SOD, GSH-Px, HG, MG, Spleen lymphocytes ↓ BUN, LDH, CK, TG, MDA, LD	[61]
Ganoderma lucidum, ‘Essence of Chicken’	Male ICR mice	833, 1666, 4165 mg/kg; 4 weeks	↑ Exhaustive swimming time, grip strength	↑ LG, MG ↓ LA, CK, BUN	[62]
Grape seed proanthocyanidin extract (GSPE)	Male ICR mice	1, 50, 100 mg/kg/day; 28 days	↑ Exhaustive swimming time	↑ SOD, CAT, SDH, Na^+^-K^+^-ATPase ↓ LA, CK, LDH, MDA, TNF-α, IL-1β	[63]
Green tea, isolated soy protein	Male ICR mice	5.13 g/kg; 4 weeks	↑ Exhaustive swimming time, grip strength	↑ LG, MG ↓ LA, NH_3_	[64]
Hairtail Fish (HGP)	Male BALB/c mice	500, 1000, 2000 mg/kg; 6 weeks	↑ Exhaustive swimming time, time to turn around, shuttle trips, active and passive escapes, total distance ↓ Errors	↑ LG, HG, CAT, GPX, SOD ↓ BLA, LDH, BUN, CK, MDA	[65]
Hualian No. 4 wild bitter gourd extract	Male ICR mice	1 g/kg, 2.5 g/kg/day; 4 weeks	↑ Swimming time, grip strength, ↓ Fatigue markers (LA, BUN, CK)	↑ PPARα, PPARγ pathways, glycogen storage, glucose metabolism ↓ Reduced muscle damage	[66]
Licorice	Male Sprague-Dawley rats	2.430 g/kg/day; 7 days	↑ Glycolytic processes, cholesterol, glycerolipid metabolism ↓ Fatigue, inflammation	↑ ATP, enzyme activity, HK2, PFKM, HMGCR, CYP7A1, GPAT, LIPIN, PFK-1, AMPK, STAT, SOD ↓ NF-κB	[67]
*Ludwigia octovalvis* (Jacq.) raven extract	Male ICR mice	0, 61.5, 307.5 mg/kg/day; 4 weeks	↑ Muscle glycogen storage, endurance performance ↓ Fatigue, muscle damage	↑ Glucose utilization, AMPK pathway ↓ Serum levels of LA, NH_3_, CK, BUN, ROS	[68]
Melon concentrate	Male Sprague Dawley rats;	16 USOD/day; 5 days in rats; 40 mg/day	↑ Fatigue resistance ↓ Muscle damage, oxidative stress, CRP	↑ Antioxidant activity (SOD), PGC-1α, mitochondrial function, muscle fibrosis reduction ↓ TNF-α	[69]
*Nelumbinis Stamen*	Mice	0.5 g, 1 g/kg/day; 15 days	↑ Antioxidant defense ↓ Muscle dysfunction, fatigue, stress hormone	↑ Sesterin2, mitochondrial homeostasis, ATP, SOD ↓ mTORC1, serum corticosterone, ROS	[70]
*Pinus koraiensis* leaf	Male ICR mice	100 mg/kg; 10 days	↑ Swimming time ↓ Latency to fall	↑ LG, MG, SOD, GSH-Px, BDNF, pCREB ↓ LA, LDH, ALT, AST, BUN, CK, serotonin, corticosterone	[71]
*Pisum sativum* L.	Kunming mice	100, 200, 400 mg/kg; 30 days	↑ Swimming time	↑ HG, LG, insulin, LDH, SOD, GSH-Px, Phagocytosis, sIgA ↓ BUN, BLA, MDA, TNF-a	[72]
*Polygonatum cyrtonema* Hua	C57BL/6 mice	65 mg, 260 mg/kg/day; 4 weeks	↑ Muscle energy metabolism and ATP production	↑ Exhaustive swimming time, liver and muscle glycogen, osteocalcin, SOD, GSH-PX ↓ Lactic acid, BUN, MDA	[73]
*Polygala tenuifolia*	Mice	0.40 mg/g/day	↑ Swimming time	↑ Antioxidant ↓ LDH	[74]
*Polygonati rhizoma* and Notoginseng Radix et Rhizoma	Male Kunming mice	1 or 2 g/kg/day; 4 weeks	↑ Swimming time, liver glycogen stores, serum biochemical markers ↓ Fatigue	↑ SOD, GSH-PX ↓ MDA, BUN, LDH	[75]
*Rhodiola Crenulata*	Male ICR mice	1.02, 3.03, 6.06 mL/kg/day; 2 weeks	↑ Antioxidant capacity, energy production, modulates mitophagy ↓ Oxidative stress	↑ SOD, GSH-PX, ATP, PINK1/Parkin ↓ LA, NH_3_, CK, pH	[76]
*Rhodiola sacra*	Male C57BL/6J mice	0.1 mL/10 g; 5 weeks	↑ Exhaustive swimming time	↑ Citrate synthase, AMPK/PGC-1α, LC3 II/LC3-I, BNIP3, DRP1, MFN1, MnSOD ↓ p62, CK, MDA	[77]
*Rubus coreanus*	ICR Mice	1 g/kg/day; 28 days	↑ Swimming time, muscle glycogen ↓ Fatigue	↑ NEFA ↓ MDA, LA, triglycerides	[78]
*Sarcodon imbricatus*	Male Kunming mice	0.25, 0.5, 1.0 g/kg; 18, 32 days	↑ Exercise time in FST, RRT ↓ Immobility duration time in TST	↑ LG, ATP, SOD, GSH-Px Nrf2, SOD1, SOD2, HO-1, CAT ↓ LDH, BUN, MDA, ROS	[79]
*Schisandra chinensis* extract (SCE)	Male Kunming mice	100, 200, 300 mg/kg; 28 days	↑ Exhaustive swimming time	↑ LG, MG, SOD, CAT, GSH-Px AMPK/PGC-1α, Nrf2, HO-1, NQO1 ↓ LA, BUN, MDA	[80]
*Sonchus arvensis* L.	Male C57BL/6 mice	250, 500 mg/kg; 4 weeks	↑ Swimming time	↑ Hemoglobin, HG, SOD, Gpx-1 ↓ Hind limb muscle length, BLA, BUN, MDA, liver PEPCK	[81]
SRB supplementation	C57BL/6 Mice	615 mg, 1230 mg, 2460 mg/kg BW; 4 weeks	↑ Exercise performance, liver and muscle glycogen storage, antioxidant enzyme activities ↓ Exercise-induced fatigue	↑ Nrf2, ARE-dependent gene, SCFA producing bacteria, *Lactobacillus*, *Bifidobacterium* ↓ MDA, quadriceps femoris morphology, sulfate-reducing bacteria	[82]
*Tremella* extract	Male, female Kunming mice	0.5 g/kg, 1.5 g/kg, 3.0 g/kg; 14 days	↑ Swimming time	↑ SOD, GSH-Px, ATP, muscle and liver glycogen levels, fatigue resistance, hypoxia tolerance	[83]
Clinical Studies					
American ginseng	Male college students (14)	4 × 400 mg/capsule; 30 days	-	↓ CK, 8-iso-PGF2 α, IL-4	[84]
Chlorella	Healthy male (27)	6 g/day; 4 weeks	F-VAS less increased	↑ Total antioxidant capacity (TAC) ↓ MDA	[85]
Coffee	Healthy endurance-trained men (14)	Coffee + milk + sucrose; 0, 60, 120 min	-	↑ MG, total AUC of insulin and glucose	[86]
Green tea extract	Untrained men	500 mg/day; 15 days	-	↓ CK	[87]
*Gynostemma pentaphyllum* extract	Healthy adults	450 mg of GPE; 12 weeks	↑ VO_2 max_ value, O_2_ pulse value, eNOS level in recovery period	-	[88]
Lemon Verbena Extract (Planox^®^)	Male 19, female (1)	400 mg/day; 10 days	↓ Muscle stiffness, muscle pain	↑ GSH-Px ↓ CK, IL-6, 8-OHdG	[29]
Lemon verbena extract (Recoverben^®^)	Healthy male (19), female (25)	400 mg/day; 15 days	↓ Muscle damages ↑ Faster recovery	↑ GPxP ↓ VAS	[89]
Mango leaf extract Combination with Quercetin	Healthy male (33), female (24)	140 mg Zynamite^®^ + 140 mg quercetin, 24 h, 4 doses	↑ Jumping performance ↓ Muscle soreness, CK, ALT	↑ Mitochondrial function, antioxidant/anti-inflammatory ↓ ROS, TNF-α inhibition	[90]
Matcha green tea beverage	Healthy male (36)	1.5 g/2 times/day; 12 weeks	↑ Muscle strength, mass, gut health ↓ Fatigue, stress	↑ Glutamate, SCFAs, amino acids, mTOR ↓ Cortisol, ROS, TNF-α, IL-6, NF-kB	[91]
Melon concentrate	Male adults (41)	40 mg/day; 8 weeks	↑ Fatigue resistance ↓ Muscle damage, oxidative stress, CRP	↑ Antioxidant activity (SOD), PGC-1α, mitochondrial function, muscle fibrosis reduction ↓ TNF-α	[69]
New Zealand Blackcurrant Extract	Healthy adults (27)	1 capsule/day; 12 days	↓ Muscle soreness, faster recovery of baseline MVC	↓ CK	[92]
*Schisandra Chinensis* extract	Postmenopausal middle-aged healthy women (65)	1000 mg/day; 12 weeks	↑ Quadriceps muscle strength	↓ LA	[93]
Shilajit	Male recreational athletes (63)	250 mg/day (low), 500 mg/day (high); 8 weeks	↑ Fatigue resistance, strength retention ↓ Collagen degradation	↑ ATP availability, mitochondrial function, antioxidant activity	[94]
Tart cherry extract (NordicCherry^®^)	Male (13)	500 mg/day; 7 days	↑ Handgrip strength, ↓ Muscle soreness	↓ Protein carbonyl, CK, CK-MB	[95]
*Tribulus terrestris*	Male CrossFit athletes (30)	770 mg/day; 6 weeks	-	↑ TAS ↓ LDH, CRP	[96]
*Zingiber officinale*	Female (50)	1 g/day,4 weeks	↓ Fatigue	↓ LDH	[97]

Arrow indicates ↑ Increase/upregulation. ↓ Decrease/downregulation.

**Table 3:** Anti-muscle fatigue effect by decoctions.

Natural Product	Animal Model/RCT(M/F)	Dose and Duration	Efficacy	Mechanism	Ref.
**Preclinical Studies**					
*Astragalus* polysaccharide	Male ICR mice	0.7, 1.4, 3.5 g/kg BW/day; 6 weeks	↑ Forelimb grip strength, swimming time, fatigue markers	↑ Glycogen storage (liver, muscle), LA, CK clearance, reduced post-exercise muscle damage	[98]
*Antrodia camphorata*, *Panax ginseng*	Male ICR mice	0.984, 2.952, 5.904 g/kg; 28 days	↑ Exhaustive swimming time, grip strength	↑ MG ↓ LA, NH_3_, CK, BUN	[99]
Danggui Buxue Tang	Male Kunming mice	5.41, 10.82, 21.64 g/kg; 15 days	↑ Swimming time, ↓ BLA, BUN	TCA cycle regulation, ↑ amino acid metabolism LA clearance ↓ oxidative stress	[100]
Liuwei Dihuang Decoction) or Yukmijihwangtang (YJT)	Male ICR mice	1, 10, 100 mg/kg/day;	↑ Endurance performance ↓ Fatigue	↑ HSP70 ↓ NE, 5-HT, 5-HIAA, DA	[101]
*Polygonati rhizoma*	Male Kunming mice	250 mg/kg/day; 14 days	↑ Swimming time, ↓ Fatigue	↑ Antioxidant activity, gut microbiota regulation ↓ LA, BUN, CK	[102]
Shuyu decoction (SYD)	Male SD rats	27.8 g/kg/day; 3 weeks	↑ Tensile force	↑ CD3^+^, CD4^+^, CD8+ ↓ LA, BUN, CK, T: R, IL-6, IL-1β, TNF-α, IL-17	[103]
**Clinical Studies**					
Jakyakgamcho-tang	Healthy male adults (30)	3 g/day; 3 days (after DOMS induction)	↑ Pain threshold ↓ Muscle soreness, calf circumference	Glycyrrhetic acid, paeoniflorin: relaxation of muscle fibers, reduction of potassium ions in myofibers	[104]

Arrow indicates ↑ Increase/upregulation. ↓ Decrease/downregulation.

**Table 5:** Activation of muscular strength via natural products.

Natural Product	Single Compound/Herb/ Decoction	Animal Model/RCT(M/F)	Dose and Duration	Efficacy	Mechanism	Ref.
Preclinical studies						
*Astragalus membranaceus*	Herb	In vitro: C2C12	10 ng/mL; 72 h	↑ Myotube diameter, muscle hypertrophy ↓ Muscle atrophy-related conditions	↑ PI3K/Akt/mTOR, muscle protein synthesis, hypertrophy	[138]
*Achyranthes bidentata*	Herb	In vitro: C2C12 In vivo: mice	10 µg/mL; 6 days; 140 mg/kg/day; 21 days	↑ Myotube diameter, muscle fiber diameter	↑ PI3K/Akt/mTOR	[139]
Cordycepin	Single compound	In vitro	10 µM, 20 µM; 1, 3, 5, 7 days	↑ Energy reserve ↓ Muscle cell differentiation, oxidative stress	↑ ERK1/2 MAPK signaling pathway ↓ Myogenic differentiation marker	[140]
*Epimedium* *(Icariin)*	Herb	In vitro: C2C12	100 µg/mL; 24 h	↑ Myotube hypertrophy, MyHC	↑ IGF-1R, PI3K/Akt/mTOR ↓ Myostatin	[141]
*Ganoderma lucidum*	Extract	Male Sprague-Dawley rats	(1) 50 mg/kg/day (2) 100 mg/kg/day (3) 200 mg/kg/day (4) 500 mg/kg/day; 6 h during hypobaric hypoxia exposure	↑ Muscle mass, antioxidant activity, muscle regeneration, myogenesis markers (Myf5, MyoD, MyoG), BDNF, IL-6 ↓ Oxidative stress markers (ROS, MDA), muscle atrophy indicators, TNF-α, MSTN	↑ Myogenesis regulatory factors (Myf5, MyoD, MyoG), BDNF and IL-6 ↓ Oxidative stress, ROS, lipid peroxidation markers, TNF-α and MSTN	[142]
Grilled nux vomica	Herb	Female Lewis rats	(1) 75 mg/kg/day (2) 150 mg/kg/day (3) 225 mg/kg/day; 4 weeks	↑ Muscle strength, holding power ↓ Clinical severity scores, inflammatory marker (IFN-γ, IL-17, TNF-α), autoantibody levels (AChR-ab)	↑ Anti-inflammatory mediators (TGF-β1) ↓ TLR-4/NF-κB signaling pathway, pro-inflammatory cytokines	[143]
*Lepidium meyenii* (Maca)	Herb	In vitro: C2C12 In vivo: ICR mice	0.10 mg/mL; 24 h 1 g/kg/day; 4 weeks	↑ Grip strength ↓ BLA, BUN	↑ Mitochondrial biogenesis ↓ ROS	[144]
Mistletoe	Herb	In vitro, In vivo (male ICR mice)	In vitro: 100 µg/mL; 16 h In vivo: (1) 400 mg/kg (2) 1000 mg/kg; 2 weeks	↑ Muscle mass, grip strength ↓ Muscle atrophy markers (Atrogin-1, MuRF1)	↑ PI3K/Akt signaling pathway ↓ FoxO signaling	[145]
*Panax ginseng* berry, soluble whey protein hydrolysate	Herb	Male C57BL/6J mice	(1) 700 mg/kg/day (2) 900 mg/kg/day (3) 1100 mg/kg/day; 8 weeks	↑ Muscle mass, grip strength, mitochondrial biogenesis ↓ Muscle atrophy, protein degradation, systemic inflammation	↑ PI3K/Akt/mTOR signaling pathway, SIRT1/PGC-1α activation, IL-10	[146]
*Rhaponticum carthamoides*, *Rhodiola rosea*	Herb	Male Wistar Han rats	250 mg *Rhaponticum carthamoides*, 250 mg *Rhodiola rosea*/day; 4 weeks	↑ Mechanical power output, type I/type II muscle fiber ratio	↑ Muscle protein synthesis	[147]
Vine tea extract (VTE)	Extract/single compound	Male ICR mice	(1) 18.75 mg/kg/day (2) 37.5 mg/kg/day (3) 75 mg/kg/day; 7 days	↑ Exhaustive swimming time, muscle mass, lean-to-fat ratio, glycogen levels, fatigue ↓ Blood lactic acid, LDH, serum urea nitrogen, CK	↑ Mitochondrial biogenesis, energy metabolism ↓ Expression of muscle atrophy-related genes	[148]
*Withania somnifera*	Herb	Male Sprague Dawley rats	500 mg/kg; 60 days	↑ Grip strength, muscle mass ↓ Inflammatory markers (IL-6, TNF-α), oxidative stress marker	↑ Antioxidant activity, anti-apoptotic marker (Bcl-2) ↓ AMPK levels, pro-apoptotic marker Bax	[149]
Clinical studies						
Blackcurrant	Herb	Male (11) Female (11)	600 mg/day; 7 days	↑ Fat oxidation ↓ Post-exercise hypotension, carbohydrate oxidation	↑ eNOS, ↓ Vasodilation and blood flow, oxidative stress	[150]
Caffeine	Compound	Participants (19)	200 mg/day; 10 min	↑ Peak concentric power, peak eccentric power, average power, total work	-	[151]
Caffeine	Compound	Female (18)	(1) CMR: 240 mg/day (2) CG: 100 mg/day	(1) CMR: ↑ Agility, hand-eye coordination, isometric hand strength, service accuracy, forehand drive, hand movement speed, movement speed, lower body explosive power (2) CG: ↑ Agility, hand movement speed, lower body explosive power, accuracy in the service test ↓ Errors in cognitive tests	↑ Plasma catecholamine ↓ Phosphodiesterase	[152]
*Chlorophytum borivilianum*	Herb	Male (41) Female (19)	3 g/day; 60 days	↑ Walking distance, handgrip strength, exercise speed ↓ Diastolic blood pressure, heart rate	↑ Antioxidant effects, cardiovascular function ↓ Stress-related physiological response	[153]
Citrus flavonoid extract (CFE)	Herb	Male (52) Female (40)	400 mg/day; 8 weeks	↑ Anaerobic power output, endurance, peak power, power performance ↓ LA production, muscle fatigue marker	↑ Nitric oxide (NO) production, vasodilation, oxygen and nutrient delivery to muscles ↓ ROS, inflammation	[154]
Essential amino acids (EAA), Tea catechins (TCCs)	Single compound	Participants (54)	540 mg/day; 24 weeks	↑ Skeletal muscle mass, knee extension strength, gait speed, quality of life	↑ Muscle protein synthesis ↓ Muscle protein degradation	[155]
Fermented red clover	Herb	Females (10)	120 mL/day; 14 days	↑ Protein aggregation ↓ Muscle atrophy	↑ Estrogen receptor β, muscle protein synthesis markers (HSP27) ↓ Muscle protein degradation markers (FOXO1, FOXO3a)	[156]
Mango leaf	Herb	Male (26) Female (24)	140 mg; 1 day	↑ Peak power output during repeated sprint exercises ↓ BLA	↓ Oxidative stress, ROS production	[157]
Marmalade	Compound	Male (30)	-	↑ Muscle power, ergogenic activity, phosphocreatine resynthesis, muscle performance ↓ Muscle soreness, fatigue	↑ Calcium release and reuptake in muscle	[158]
*Rhodiola rosea*	Herb	Male (10)	2000 mg/day; 3 days	↑ Bench press velocity ↓ Total repetitions to failure	↑ Norepinephrine levels	[159]
Spinach	Herb	Male (8) Female (37)	2 g/day; 12 weeks	↑ Knee extension torque ↓ Fat mass	↑ PI3K/Akt pathway, protein synthesis, energy metabolism	[160]
Whey	Single compound	Older adults (115)	32.4 g/day; 12 weeks	↑ Handgrip strength, gait speed, and chair-stand performance ↓ Fatigue and muscle weakness	↑ Muscle protein synthesis, bioavailability of branched-chain amino acids	[161]
*Withania somnifera*	Herb	Male (40)	500 mg/day; 12 weeks	↑ 1-RM squat, bench press, power and endurance ↓ Muscle soreness, post-exercise fatigue	↑ Protein synthesis, muscle damage, recovery, neuromuscular function ↓ Oxidative stress	[162]

Arrow indicates ↑ Increase/upregulation. ↓ Decrease/downregulation.

**Table 6:** Effects of natural products on muscular nervous system activation.

Natural Product	Single Compound/Herb/ Decoction	Animal Model/RCT(M/F)	Dose and Duration	Efficacy	Mechanism	Ref.
Preclinical Studies						
Yeast-derived peptides	Peptide	Male ICR mice	10, 25, 50 mg/kg; 4 weeks	↑ Exercise endurance (treadmill time) ↓ Muscle damage, exercise-induced fatigue	↑ AMPK phosphorylation ↓ LA, urea nitrogen	[163]
Clinical Studies						
*Camellia sinensis*	Single compound	Male (38)	2 g/day; 28 days	↑ Faster muscle recovery ↓ Muscle cell damage	↓ BAD, Caspase 3, 8, 9 ↑ JNK, Bcl-2	[164]
*Cordyceps sinensis*	Herb	Male (17), Female (13)	2 g/day; 12 weeks	↑ Aerobic performance, 5 km run time ↓ Heart rate at the same submaximal intensity	↑ AMPK, PGC-1α ↓ Free radical production	[165]
Nanobubble water curcumin	Single compound	Female (12)	15 g (230.9 mg curcumin)/day; 28 days	↑ Ground contact time during drop jumps ↓ Peak vertical ground reaction force in drop jumps, injury risk	↑ Glucose utilization, HDL ↓ ALT, ALP, TG, LA, NH_3_	[166]

Arrow indicates ↑ Increase/upregulation. ↓ Decrease/downregulation.

**Table 7:** Anti-inflammatory effects from natural products.

Natural Product	Single Compound/Herb/ Decoction	Animal Model/RCT(M/F)	Dose and Duration	Efficacy	Mechanism	Ref.
Black Chokeberry Extract	Herb	Male football players (22)	6 g/90 days (7 times per week)	↑ Performance	↓ IL-6, IL-10 (inflammatory markers)	[167]
Coffee	Single compound	Resistance-trained athletes (15)	6 mg/kg/1 h before exercise	↓ Inflammatory responses	↑ TT genotype	[168]
Dipeptide Extract	Single compound	Male (20)	4 g/14 days	↓ Oxidative stress, inflammation	↑ NO/H_2_O_2_ ↓ C-reactive protein levels	[169]
Fucoidan	Decoction	Male (8), Female (8)	1 g/day/2 weeks	↑ Immune response ↓ Inflammation	All blood markers or performance outcomes	[170]
Nopal Cactus Fruit Juice	Herb	40 (each 20) either gender	3 ounces/daily 8 weeks	↑ ROM ↓ Pain daily activities	↓ CRP, Eotaxin, serum cytokine	[171]

Arrow indicates ↑ Increase/upregulation. ↓ Decrease/downregulation.

**Table 8:** Additional physiological benefits of natural products.

Natural Products	Single Compound/Herb/ Decoction	Animal Model/RCT(M/F)	Dose and Duration	Efficacy	Mechanism	Ref.
Preclinical studies						
Ashitaba (*Angelica keiskei*)	Single compound	Male ICR mice	250, 500 mg/kg/day; 28 days	↑ Body weight, muscle thickness	↑ MyoD, myogenin ↓ MuRF1, Atrogin-1/MAFbx	[172]
Cocoa flavanols	Single compound	Male 129S1/Svlmj mice	302.1 mg/kg body weight twice a day; 15 days	↑ Mitochondrial function, whole-body metabolism	↑ NAD^+^, NADH, Sirt3, mitochondrial mass	[173]
Cinnamon	Decoction	Rat	200 mg/kg body weight	↑ Insulin sensitivity ↓ HbA1c, TBC1D1, TBC1D4	↑ GLUT4, glucose uptake, mitochondrial efficiency, brown adipose tissue activity	[174]
Green tea catechins	Single compound	Caenorhabditis elegans	2.5 µM; Lifespan	↑ Lifespan, motility ↓ Age-associated pigment	↑ ROS, DAF-16, SKN-1/Nrf2 ↓ Mitochondrial complex I activity	[175]
Hesperidin	Single compound	Female rats	200 mg/kg BW 3 times/week; 5 weeks	↑ IgA	↑ T cell, NK cell ↓ Cytokines, B cell	[176]
Juzentaihoto	Decoction	Mice	4% JTT-mixed feed; 56 days	↑ Metabolism ↓ Skeletal muscle atrophy, inflammation	↑ Sirtuin1, adiponectin ↓ Insulin resistance, TNF-α and IL-6, HOMA-R, ubiquitin ligases	[177]
Lemon myrtle (Casuarinin)	Herb	Male Sprague-Dawley rats	LM (250 mg/kg/day), casuarinin (4 and 8 mg/kg/day); 4 days	↑ Satellite cells	↑ IL-6, mRNA expression	[178]
*Salvia plebeia* R.Br.	Single compound	In vitro	SPR 10 μg/mL or RosA 5 μM for 24 h	↓ Atrophy	↑ SOD activity, mitochondrial function, Akt/mTOR/p70S6K pathway ↓ Protein degradation, ROS, autophagy, apoptosis	[179]
Shatavari supplements	Herb	Female (12)	26,500 mg/day fresh weight; 6 weeks	↑ Integration of energy metabolism, neutrophil degranulation, chemical synapse ↓ Translation, muscle contraction, viral infection	↑ Integrin/MAPK/BRAF/RAF signaling ↓ Translation/amino acid metabolism	[180]
Xiaoyao san	Herb	Male Sprague-Dawley rats (24)	10 mL/kg/day; 28 days	↑ Exercise capacity, liver mitochondrial metabolomics ↓ Depression	↑ AGE-RAGE, HIF-1, calcium signaling pathway ↓ Sugar water preference rate, lactic acid, aspartic acid, phenylalanine, and glutathione	[181]
Yiqi chuta formula	Decoction	Female C57BL/6 mice (24)	4 g/kg/day; 28 days	↑ Antioxidant ↓ Cisplatin-induced skeletal muscle damage	↓ ROS, Cleaved Caspase-3, PARP, GPX4, intracellular iron concentration, MDA	[182]
Clinical studies						
Aronia berry	Herb	RCT (70)	300 mg/day; 8 weeks	↑ Glutathione defense system, antioxidant capacity	↑ GSH, GPx ↓ ROS, oxLDL, MDA	[183]
Bay leaf	Decoction	RCT (60)	75 mL/twice a day; 7 days	↓ Blood cholesterol level	↓ Cholesterol absorption, lipid peroxidation	[184]
Black currant	Herb	Male (16)	315 mg anthocyanins; 2 h before measurement	↑ 5 km running time	↑ NO	[185]
Brazilian propolis	Single compound	RCT (F)	454 mg/day; 12 weeks	↑ Serum adiponectin ↓ Body fat mass, oxidative stress level	↑ Adiponectin, SOD ↓ ROS	[186]
Broccoli	Herb	RCT (44)	10 g/day;12 weeks	↑ Insulin sensitivity, lipid metabolism	↑ HDL ↓ ROS, MDA	[187]
Broccoli sprout	Herb	RCT (09)	150 g/day; 9 days	↑ Mitochondrial function, physical performance ↓ LA accumulation	↑ Nrf2 ↓ ROS, inflammation	[188]
Caffeinated chewing gum	Single compound	RCT (9 male, 3 female)	3.2 ± 0.4 mg/kg; 15 min before trial	↑ Attack accuracy	-	[189]
Caffeine soluble coffee	Single compound	RCT (9 male, 2 female)	3 mg/kg; 60 min before trial	↑ Mean heart rate ↓ total time	↑ Dopamine, norepinephrine, epinephrine (adrenaline) ↓ Adenosine	[190]
Coffee	Single compound	RCT (126)	Daily drinking (220 g median); 3 days	↑ Gait speed, SPPB, chair rise point	↑ Caffeine	[191]
Green tea	Herb	Female (30)	1500 mg/3 times/day; 10 weeks	↑ Metabolic responses, Antioxidant capacity	↑ VO_2 max_, SIRT1, PGC-1α, CAT ↓ Body fat percentage, BMI	[192]
L-arginine	Single compound	Male (56)	2 g/day; 45 days	↑ Sport performance, VO_2 max_	↑ Muscular power, blood flow nutrient delivery	[193]
Probiotics and vitamin D	Single compound	Male (25)	Probiotics 2 × 10^9^ CFUs Vit D 3000–4000 IU/day; 4 weeks	↑ Anaerobic performance, serum concentrations of 25(OH)D3 ↓ BLA, muscle damage	↑ Utilization of lactate, maximal oxygen intake, duration, intensity, muscle protein synthesis, mitochondrial function	[194]
Vitamin D3	Single compound	Male (46)	35,000 or 70,000 IU/week; 12 weeks	↑ Serum 25(OH)D ↓ PTH	↑ Vit D and calcium homeostasis	[195]

Arrow indicates ↑ Increase/upregulation. ↓ Decrease/downregulation.

## Data Availability

No new data were created or analyzed in this study. Data sharing is not applicable to this article.

## Abbreviations

The following abbreviations are used in this manuscript:

ACCα	Acetyl-CoA carboxylase-α
AMPK	AMP-activated protein kinase
AOPP	Advanced oxidation protein products
Arg1	Arginase 1
Ass1	Argininosuccinate synthase 1
AST	Aspartate aminotransferase
ATGL	Adipose Triglyceride Lipase
ATP	Adenosine triphosphate
Bcl-2	B-cell lymphoma-2
BDNF	Brain-derived neurotrophic factor
BLA	Blood lactate
BNIP3	BCL2/adenovirus E1B 19 K Da-interacting protein 3
BRAF	B-Raf Proto-Oncogene, serine/threonine kinase
BUN	Blood urea nitrogen
C/EBPα	CAAT/enhancer binding protein alpha
C2C12	Immortalized mouse myoblast cell line
CAT	Chloramphenicol Acetyl Transferase
CD3+	Cluster of differentiation 3
CK	Creatine kinase
Cco	Cytochrome c oxidase
Cps1	Carbamoyl phosphate synthase 1
CRP	C-Reactive Protein
CYP7A1	Cytochrome P450 Family 7 Subfamily A Member 1
CytC	Cytochrome C
DA	Dopamine
DAF-16	SKN-1/Nrf2
DGAT	Diacylglycerol-O-acyltransferase
DRP1	Dynamin-related protein 1
EDRF	Endothelium-derived relaxing factor
EPO	Erythropoietin
ERK1	Extracellular signal-regulated kinase 1
FASN	Fatty acid synthase
FOXO1	Forkhead box protein O1
GLU	Glutamate
GLUT4	Glucose transporter type 4
GPT	Glutamate pyruvate transaminase
GPX	Glutathione peroxidase
GSSG	Glutathione disulfide
GST	Glutathione S-transferases
HDL	High-density lipoprotein
HK2	Hexokinase 2
HMGCR	3-hydroxy-3-methylglutaryl-CoA reductase
HO1	Heme oxygenase 1
HOMA-R	Homeostatic Model Assessment for Resistance
HSL	Hormone-sensitive lipase
HSP70	Heat shock protein 70
IL-6	Interleukin-6
JNK	Jun N-terminal kinase
Keap1	Kelch-like ECH-associated protein 1
LA	Lactate
LC3B	Microtubule associated protein 1 light chain 3 beta
LDH	Lactate dehydrogenase
LG	Liver glycogen
LKB1	Liver Kinase B1
MAFbx	Muscle Atrophy F-box gene
MAPK	Mitogen-activated protein kinases
MDA	Malondialdehyde
MEF2C	Myocyte enhancer factor 2C
MFN1	Mitofusin-1
MG	Muscle glycogen
MnSOD	Manganese superoxide dismutase
MSTN	Myostatin
mtDNA	Mitochondrial DNA
mTORC1	Mammalian target of rapamycin complex 1
MUAC	Mid-upper-arm circumference
MuRF1	Muscle-specific RING finger protein 1
Myf5	Myogenic factor 5
MyHC-I	Myosin heavy chain type I
MyoD	Myoblast determination protein
NAD	Nicotinamide adenine dinucleotide
Nags	N-acetylglutamate synthase
NE	Norepinephrine
NF-κB	Nuclear factor kappa-light-chain-enhancer of activated B cells
NH_3_	Ammonia
NK cells	Natural killer cells
NQO1	NAD(P)H quinone dehydrogenase 1
Nrf2	Nuclear factor erythroid 2-related factor 2
NEFA	Non-esterified fatty acids
OCR	Oxygen consumption rate
Otc	Ornithine transcarbamylase
oxLDL	Oxidized low density lipoprotein
PI3K	Phosphoinositide 3-kinase
PARP1	Poly(ADP-ribose) polymerase 1
PEPCK	Phosphoenolpyruvate carboxykinase
PFK-1	Phosphofructokinase-1
PGC-1α	Peroxisome proliferator-activated receptor-gamma coactivator-1alpha
PINK1	PTEN-induced kinase 1
PPARγ	Peroxisome proliferator-activated receptor gamma
ROS	Reactive Oxygen Species
SCFAs	Short-chain fatty acids
SDH	Succinate dehydrogenase
SIRT1	Sirtuin 1
SREBP1C	Sterol regulatory element-binding protein-1c
STAT-3	Signal transducer and activator of transcription 3
TAS	Tyrosine auxotrophy suppressor
TCA	Trichloroacetic acid
TFAM	Mitochondrial transcription factor A
TG	Thyroglobulin
TGF-β1	Transforming growth factor beta 1
TGR5	Takeda G protein-coupled receptor 5
TLR-4	Toll-like receptor 4
TNF-α	Tumor necrosis factor alpha
TNNC1	Troponin C1
T:C	Testosterone to Cortisol ratio
VAS	Visual analogue scales
VO_2max_	Maximal oxygen consumption
5-HIAA	5-hydroxyindole-acetic acid
5-HT	Serotonin, 5-hydroxytryptamine
8-iso-PGF2α	8-iso Prostaglandin F2α
8-OHdG	8-Hydroxy-2′-deoxyguanosine
